# Presence of chondroitin sulphate and requirement for heparan sulphate biosynthesis in the developing zebrafish inner ear

**DOI:** 10.3389/fcell.2022.959624

**Published:** 2022-08-26

**Authors:** Ana A. Jones, Elvira Diamantopoulou, Sarah Baxendale, Tanya T. Whitfield

**Affiliations:** Development, Regeneration and Neurophysiology, School of Biosciences, and Bateson Centre, University of Sheffield, Sheffield, United Kingdom

**Keywords:** extracellular matrix, heparan sulphate proteoglycan, chondroitin sulphate proteoglycan, epithelial morphogenesis, semicircular canals, otoliths, zebrafish, Ext2

## Abstract

Epithelial morphogenesis to form the semicircular canal ducts of the zebrafish inner ear depends on the production of the large glycosaminoglycan hyaluronan, which is thought to contribute to the driving force that pushes projections of epithelium into the lumen of the otic vesicle. Proteoglycans are also implicated in otic morphogenesis: several of the genes coding for proteoglycan core proteins, together with enzymes that synthesise and modify their polysaccharide chains, are expressed in the developing zebrafish inner ear. In this study, we demonstrate the highly specific localisation of chondroitin sulphate to the sites of epithelial projection outgrowth in the ear, present before any morphological deformation of the epithelium. Staining for chondroitin sulphate is also present in the otolithic membrane, whereas the otoliths are strongly positive for keratan sulphate. We show that heparan sulphate biosynthesis is critical for normal epithelial projection outgrowth, otolith growth and tethering. In the *ext2* mutant ear, which has reduced heparan sulphate levels, but continues to produce hyaluronan, epithelial projections are rudimentary, and do not grow sufficiently to meet and fuse to form the pillars of tissue that normally span the otic lumen. Staining for chondroitin sulphate and expression of *versican b*, a chondroitin sulphate proteoglycan core protein gene, persist abnormally at high levels in the unfused projections of the *ext2* mutant ear. We propose a model for wild-type epithelial projection outgrowth in which hyaluronan and proteoglycans are linked to form a hydrated gel that fills the projection core, with both classes of molecule playing essential roles in zebrafish semicircular canal morphogenesis.

## 1 Introduction

Morphogenesis of the vertebrate inner ear is an excellent exemplar of epithelial morphogenesis. Development of this organ system involves a series of events that shape and fold the otic epithelium, converting it from a fluid-filled vesicle into a series of interlinked sensory chambers and tubes (reviewed in Alsina and Whitfield, 2017). In the zebrafish, morphogenesis of the semicircular canal ducts is initiated when finger-like protrusions or projections of epithelium move into the lumen of the vesicle. Between days 2 and 3 of embryonic development, anterior, posterior and ventral projections fuse in a stereotyped sequence with corresponding bulges from a lateral projection. These fusion events create three new columns or pillars of tissue that span the otic lumen, defining the three semicircular canals (Waterman and Bell, 1984; Haddon and Lewis, 1996).

It has long been recognised that the extracellular matrix (ECM) plays a critical role in formation and outgrowth of the epithelial projections in the zebrafish and *Xenopus* ear. Electron microscopy studies revealed that the acellular cores of the projections are filled with “fibrillar and granular material”, and that cells of the projection epithelium are rich in endoplasmic reticulum and lack a basal lamina, consistent with high levels of ECM production and secretion (Waterman and Bell, 1984; Haddon and Lewis, 1991). A major ECM component of the projection cores is the glycosaminoglycan (GAG) hyaluronan (also known as hyaluronic acid or HA) (Haddon and Lewis, 1991; Toole, 2001; Busch-Nentwich et al., 2004). Haddon and Lewis first proposed that localised synthesis and retention of this large, space-filling polymer could act as a driving force for the outgrowth of epithelial projections into the lumen of the *Xenopus* otic vesicle (Haddon and Lewis, 1991). In support of this hypothesis, localised enzymatic digestion of HA blocks projection outgrowth in both *Xenopus* (Haddon and Lewis, 1991) and zebrafish (Geng et al., 2013; Munjal et al., 2021). Moreover, reducing HA production in zebrafish, via morpholino-mediated knockdown of *dfna5* (orthologue of the human deafness autosomal dominant *DFNA5* gene) or *hyaluronan synthase* (*has3*), or genetic mutation of *UDP-glucose dehydrogenase* (*ugdh*), compromises projection outgrowth (Neuhauss et al., 1996; Walsh and Stainier, 2001; Busch-Nentwich et al., 2004; Munjal et al., 2021).

The zebrafish *ugdh* (*jekyll*) mutant otic phenotype is severe, with very small and rudimentary projections, even by 6 days post fertilisation (dpf) (Neuhauss et al., 1996). The *ugdh* gene, which is expressed in the epithelial projections of the zebrafish ear (Walsh and Stainier, 2001; Busch-Nentwich et al., 2004), is an orthologue of *sugarless* (*sgl*) in *Drosophila* (Häcker et al., 1997), and codes for the enzyme UDP-glucose 6-dehydrogenase (Ugdh). Ugdh catalyses the production of glucuronic acid, an essential building block not only of HA, but also of various proteoglycans, including heparan sulphate, chondroitin sulphate and keratan sulphate proteoglycans (HSPGs, CSPGs, KSPGs) (reviewed in Zimmer et al., 2021), suggesting that deficiencies of these molecules might also contribute to the *ugdh* mutant phenotype.

Proteoglycans are negatively charged ECM molecules consisting of sulphated GAG chains attached to a protein core, with widespread roles in animal development (reviewed in Bülow and Hobert, 2006). Chondroitin sulphate proteoglycans (CSPGs) are already implicated in zebrafish otic morphogenesis. The CSPG core protein gene *versican a* (*vcana*) is highly expressed in the epithelial projections during their growth phase, but is rapidly downregulated on fusion to form pillars (Geng et al., 2013). The zebrafish ear expresses chondroitin synthase and glycosyltransferase genes coding for enzymes involved in the polymerisation of CS chains (Li et al., 2010; Filipek-Górniok et al., 2013), together with sulfotransferase genes involved in CS modification (Habicher et al., 2015). Morpholino-mediated knockdown of *chondroitin synthase 1* (*chsy1*) leads to epithelial projection defects in the zebrafish ear (Li et al., 2010). Here, we use immunohistochemistry to show that localised deposition of CS prefigures the sites of projection outgrowth in wild-type zebrafish ears.

To test the role of heparan sulphate (HS) in zebrafish semicircular canal morphogenesis, we have examined the otic phenotype of the *exostosin 2* (*ext2/dackel*) mutant, which lacks the function of a glycosyltransferase involved in HS biosynthesis (Lee et al., 2004). Mutants have a global reduction in HS levels by 5 days post fertilisation (dpf), and form a model for the human autosomal dominant condition multiple osteochondromas (Lee et al., 2004; Clément et al., 2008; Holmborn et al., 2012). Homozygous zebrafish *ext2* mutations disrupt development of cartilage, bone and teeth (Schilling et al., 1996; Clément et al., 2008; Wiweger et al., 2012; Wiweger et al., 2014), pectoral fins (van Eeden et al., 1996), the retinotectal projection (Karlstrom et al., 1996; Trowe et al., 1996; Lee et al., 2004), and the lateral line (Venero Galanternik et al., 2015). These pleiotropic effects reflect the widespread requirement of HSPGs for the activity of various developmental signalling pathways (see, for example, Norton et al., 2005; Fischer et al., 2011; Venero Galanternik et al., 2015). Otic defects were noted in *ext2* mutants (Whitfield et al., 1996), but were not characterised in detail. We now show that *ext2* mutants have otic defects similar to those seen after disruption of HA or CS, demonstrating an essential role for HS biosynthesis in epithelial projection outgrowth and formation of the semicircular canal system. Our results indicate that the otic ECM is composed of both HA and proteoglycans, and that the loss of either class of molecule compromises the early steps of semicircular canal morphogenesis.

## 2 Materials and methods

### 2.1 Animals

**FIGURE 1:**
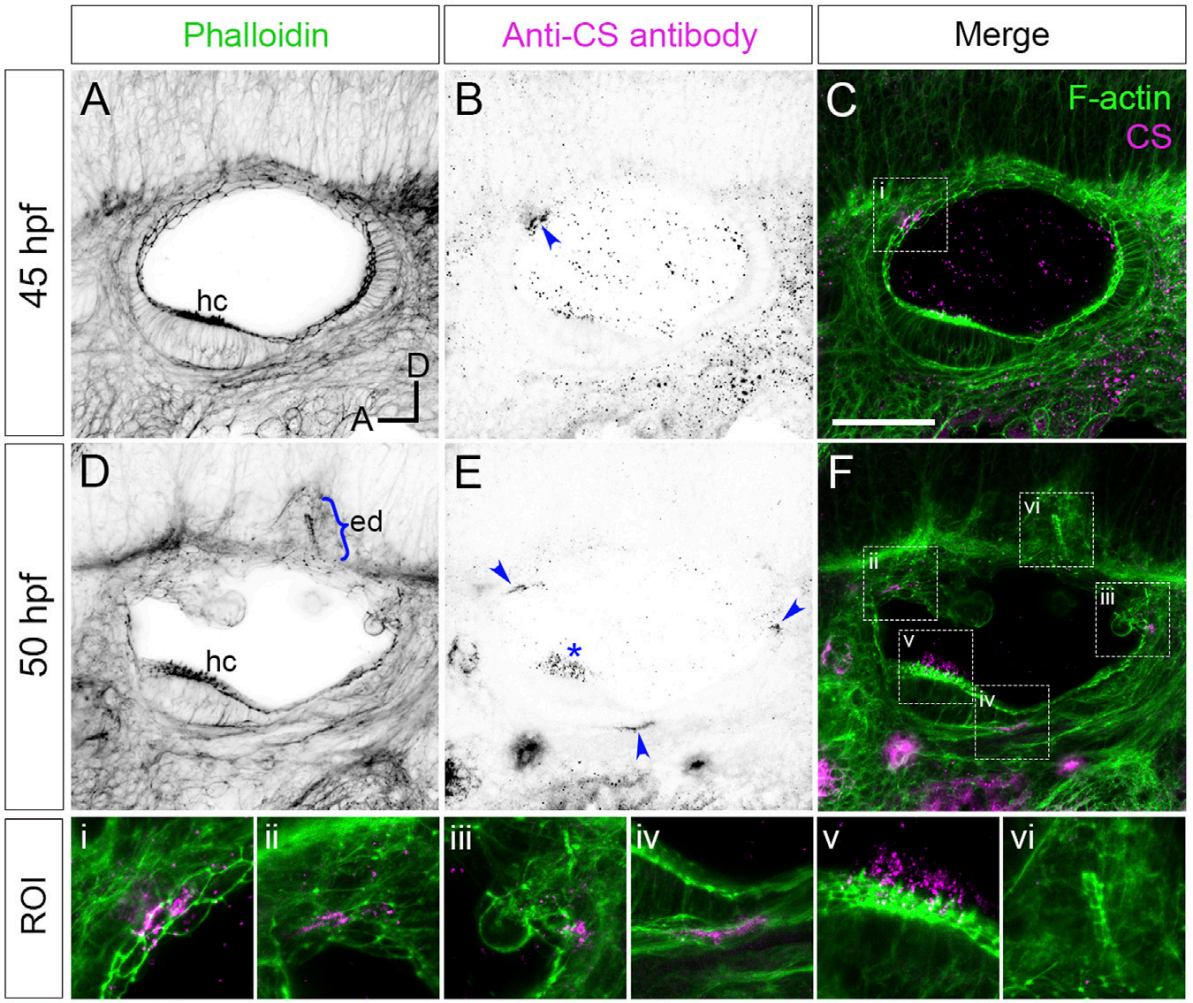
Staining for chondroitin sulphate in phenotypically wild-type zebrafish ears marks sites of epithelial projection outgrowth. **(A–F)** Confocal images of Alexa-phalloidin (green) and anti-CS antibody (magenta) whole-mount stains of phenotypically wild-type ears (Section 2). Blue arrowheads mark foci of chondroitin sulphate (CS) staining associated with the emergence (evagination) of epithelial projections from the otic epithelium; blue bracket marks the invaginating endolymphatic duct (ed); blue asterisk marks staining in the otolithic membrane overlying hair cells of the anterior (utricular) macula. Boxed areas highlight regions of interest (ROI), enlarged in the bottom row of panels: **(i)**, apical focus of CS staining at 45 hpf prefiguring site of emergence of the anterior projection; **(ii,iii)**, foci of CS staining associated with the anterior and posterior projections, respectively, at 50 hpf; **(iv)**, basal focus of CS staining prefiguring site of emergence of the ventral projection; **(v)**, CS staining in the utricular otolithic membrane; **(vi)**, absence of CS stain in the endolymphatic duct. All panels are lateral views with anterior to left, and dorsal to top (orientation shown in **(A)**: A, anterior; D, dorsal). Scale bar in **(C)**, 50 µm (applies to **(A–F)**). Abbreviations: CS, chondroitin sulphate; ed, endolymphatic duct; hc, stereociliary bundles on the apices of hair cells in the anterior (utricular) macula. Additional examples and developmental stages are shown in Figure 4; Supplementary Figure S1; Supplementary Movie S1.

Standard zebrafish husbandry methods were employed (Westerfield, 2007; Aleström et al., 2020). The wild-type strain used was AB (ZDB-GENO-960809-7), and the *ext2* mutant allele used was *dak* ^*tw25e*^ (ZDB-ALT-980203-1459) (Haffter et al., 1996). This allele has a T >A mutation in exon 5, introducing a STOP codon and predicting a truncation of the protein sequence at amino acid 227 (Lee et al., 2004). In all examples shown, mutant embryos were homozygous for the zygotic *dak* ^*tw25e*^ allele. For some experiments, fish were crossed onto a *nacre* (*mitfa* ^*−/−*^; ZDB-GENO-990423-18) background, which has reduced body pigmentation, facilitating visualisation of staining patterns (Lister et al., 1999). The phenotypically wild-type sibling embryos shown in Figures 1, 4E,E’, were from a cross between parents heterozygous for mutations in *tbx1* ^*tm208*^ (ZDB-ALT-980203-1362). Embryos were raised in E3 embryo medium (5 mM NaCl, 0.17 mM KCl, 0.33 mM CaCl_2_, 0.33 mM MgSO_4_) (Westerfield, 2007) at 28.5°C. In some cases, to obtain the desired stage, embryos were raised at temperatures ranging from 23°C to 33°C, and staged according to the equivalent hours post fertilisation (hpf) at 28.5°C using a conversion formula (Kimmel et al., 1995). Methylene blue (0.0001%) was added to reduce fungal growth, and where necessary, 0.003% 1-phenyl 2-thiourea (PTU; 0.003%) was added to block the development of pigmentation. We use the term embryo throughout to refer to zebrafish embryos and larvae from 0 to 5 days post fertilisation (dpf).

### 2.2 Genotyping

The *dak* ^*tw25e*^ mutation introduces an *Mse*I restriction site into the genomic DNA sequence, allowing the identification of mutant embryos at early stages [up to 48 h post fertilisation (hpf), before the onset of any obvious phenotype] by genotyping. Forward (5’-GGC​TTC​TCC​ACA​TGG​ACC​TA-3’) and reverse (5’-CCG​AGG​ACT​GGA​AGA​AAA​AC-3’) primers were used to amplify genomic DNA from individual embryos by PCR. The amplified product (188 base pairs, bp) was digested with *Mse*I (New England Biolabs, R0525S), which cuts the mutant sequence to generate two fragments of 158 bp and 30 bp.

### 2.3 Immunofluorescence and phalloidin staining

Embryos were dechorionated and fixed at the desired stages in 4% paraformaldehyde for 2–4 h at room temperature (except 40 hpf embryos, which were fixed overnight at 4°C). Fixed embryos were permeabilised in PBS-Tr (phosphate-buffered saline (PBS),1% Triton X-100) for 3 × 5 min, blocked in Blocking Solution (PBS-Tr with the addition of 10% sheep or donkey serum, depending on the secondary antibody used) for 60 min, and incubated overnight at 4°C in Blocking Solution and 1% DMSO, with the addition of the relevant primary antibody at the following concentrations: anti-Chondroitin Sulphate (CS) (C8035 mouse monoclonal, Sigma-Aldrich), 1:100; anti-Keratan Sulphate (KS) (3H1 mouse anti-rat monoclonal, R. U. Margolis and R. J. Margolis, Developmental Studies Hybridoma Bank), 1:100. Embryos were washed in PBS-Tr for 3 × 5 min and 4 × 30 min, and then incubated overnight at 4°C in Blocking Solution and 1% DMSO with the addition of an Alexa-647-conjugated goat anti-mouse secondary antibody (A21235, Invitrogen; 1:200). Embryos were counterstained for F-actin by the addition of Alexa Fluor 488 Phalloidin (8878, Cell Signalling Technology, 1:100 from a 6.6 µM stock) to the secondary antibody staining step. Embryos were washed in PBS-Tr for 4 × 5 min, and stored in PBS-Tr at 4°C before mounting. Staining with the secondary antibody only gave no signal in the ear (not shown). As an additional control, embryos from the same batch were stained with primary antibodies to CS or KS in the same experiment, using the same secondary antibody. Each primary antibody gave a different and reproducible staining pattern (Supplementary Figure S1). Staining experiments were performed in duplicate, and at least two embryos were imaged for each genotype and stage.

### 2.4 Hyaluronic acid binding protein staining

The protocol for HABP staining was adapted from a published method (Munjal et al., 2021). Embryos were dechorionated at 57 hpf and fixed in 4% paraformaldehyde at room temperature for 2 h. Embryos were washed in PBS for 3 × 5 min, before permeabilisation with ice-cold acetone at −20°C for 7 min. Embryos were washed in PBS-Tr for 3 × 5 min and blocked in PBS-Tr containing 5% BSA for 60 min at room temperature. Embryos were incubated in blocking solution containing biotinylated Hyaluronan Binding Protein (HABP from EMD Millipore, 1:100 dilution of 0.5 μg/μL stock) at 4°C, overnight. Embryos were washed 3 × 15 min with PBS (detergent was avoided due to low binding affinity of HABP to HA). Embryos were incubated with Streptavidin Alexa Fluor 546 (Invitrogen S11225, 1:500) and counterstained for F-actin with Alexa Fluor 488 Phalloidin (8878, Cell Signalling Technology, 1:100 from a 6.6 µM stock). Embryos were washed for 3 × 15 min with PBS before mounting for imaging.

### 2.5 Whole-mount *in situ* hybridisation

**FIGURE 2:**
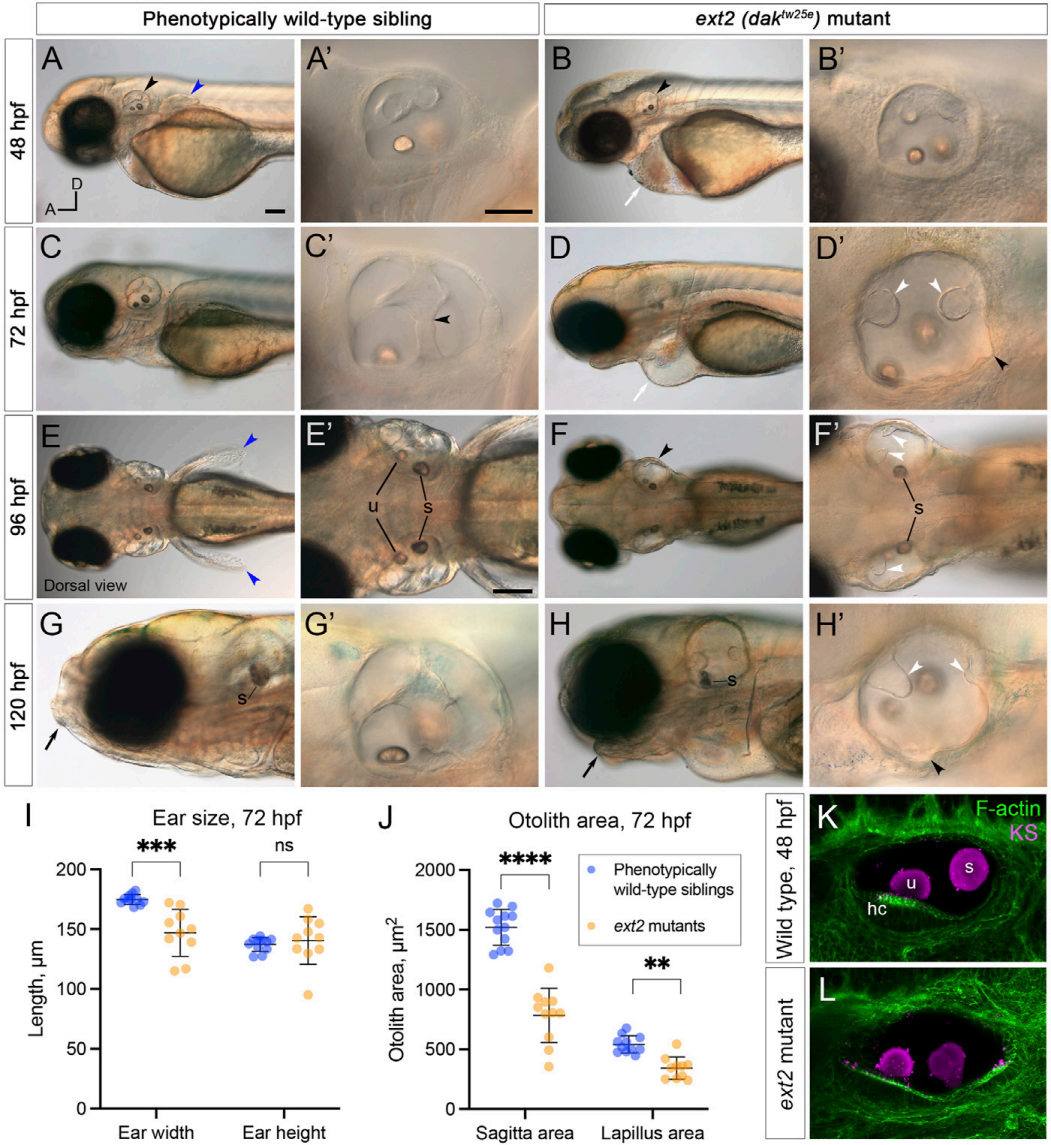
Morphological defects in the inner ear of *ext2* mutant embryos. **(A–H’)** Live DIC images of phenotypically wild-type sibling and *ext2* homozygous mutant embryos at 48–120 hpf. The otic vesicle (enlarged in the second and fourth columns) is marked with a black arrowhead in **(A,B,F)**. Note the absence of the pectoral fin bud [**(A,E)**, blue arrowheads in wild type], cardiac oedema [**(B,D)**, white arrows], and abnormal jaw [**(G,H)**, black arrows] in the mutant. At 72 hpf, epithelial projections have fused and formed pillars in the wild-type ear [black arrowhead in **(C’)** marks the fusion plate of the ventral pillar], whereas in the *ext2* mutant ear, the projections stayed small and often did not fuse [white arrowheads, **(D’,F’,H’)**]. Black arrowheads in **(D’)** and **(H’)** mark abnormal out-pocketings of the epithelium. Scale bars: **(A)**, 100 μm [also applies to **(B,C,D,E,F,G,H)**]; **(A’)**, 50 μm [also applies to **(B’,C’,D’,G’,H’)**]; **(E’)**, 100 μm [also applies to **(F’)**]. All panels are lateral views with anterior to the left (orientation shown in **(A)**: A, anterior; D, dorsal), apart from **(E–F’)** (dorsal views, anterior to the left). **(I,J)** Morphometric measurements of ear width and height **(I)**, and otolith area **(J)**, traced from micrographs at 72 hpf [*N =* 6 embryos, *n* = 12 ears of each genotype (some data points missing)]. See Supplementary Figure S2 for details. Horizontal bars show mean +/− standard deviation. Two-way ANOVA (mixed-effects model) with Šídák’s correction for multiple comparisons: ns, not significant; **p = 0.0082; ***p = 0.0008; ****p < 0.0001. **(K,L)** Ears at 48 hpf, stained with Alexa phalloidin (green) and an antibody to keratan sulphate (magenta). Abbreviations: hc, hair cells in the utricular macula; s, saccular otolith (sagitta); u, utricular otolith (lapillus).

Embryos were dechorionated and fixed in 4% paraformaldehyde overnight at 4°C. Fixed embryos were rinsed in PBS, dehydrated through a methanol series, and stored in methanol at −20°C. Whole-mount *in situ* hybridisation to the genes listed in Supplementary Table S1 was performed based on the standard protocol (Thisse and Thisse, 2008) with minor modifications. Data shown in Figure 2 are representative of two individual experiments for each gene (*n* = 50 embryos analysed per embryonic stage).

### 2.6 Microscopy and photography

For live imaging, zebrafish embryos were anaesthetised in 4% (w/v) tricaine (MS222) in E3 medium and mounted in 3% (w/v) methylcellulose in a cavity created by cutting a small window in three layers of electrical insulation tape stuck on a glass slide, and coverslipped. For immunofluorescence, fixed embryos were dissected to remove the yolk and eyes, and then mounted laterally in 1–1.5% Low Melting Point Agarose in PBS in the centre of a 35-mm Wilco glass-bottomed Petri dish. Image stacks (*z-*step, 1 µm) were acquired on a Nikon A1 confocal microscope with a × 40 objective using the 488 and 647 nm excitation laser lines. HABP-stained embryos were mounted in low-melting point agarose in glass capillaries and imaged using a Zeiss Z1 light-sheet microscope. Image stacks were acquired with a ×20 objective using the 488 and 561 excitation laser lines. Images were taken dorsally with the embryo rotated by 25° to obtain the best view of the acellular core of the lateral projection in the ear.

Images of live embryos and those stained using *in situ* hybridisation were acquired on an Olympus BX-51 compound microscope equipped with a C3030ZOOM camera and CELL B software, or a Micropublisher 6 camera and Ocular software. Images for the measurements shown in Figure 2; Supplementary Figure S2 were acquired on a Zeiss Axio Zoom. V16 microscope. Unless otherwise stated, embryos were oriented laterally, with anterior to the left. Single channel images are displayed in inverted grayscale. Images were processed in Fiji (Schindelin et al., 2012), and figures were assembled with Adobe Photoshop v22.5.1.

### 2.7 Otolith displacement (“tapping”) assay

Anaesthetised *ext2* mutants (*N* = 8) and phenotypically wild-type siblings (*N* = 8) at 5 dpf were individually mounted in 4% methylcellulose in a cavity made by cutting a window in four layers of electrical insulation tape on a microscope slide, and covered with a coverslip. The slide was then gently tapped on the benchtop for 10 s. Images were taken before and after tapping. This protocol was adapted from an earlier study, where it was used to measure otolith tethering in zebrafish mutants for *otogelin* and *tecta* (Stooke-Vaughan et al., 2015)*.*

### 2.8 Measurements and statistical analysis

Ear and otolith morphometric measurements were made from 2D micrographs in Fiji (Schindelin et al., 2012). Data were analysed and presented using GraphPad Prism v9.1.2. Statistical tests used are stated in the relevant figure legend.

## 3 Results

### 3.1 Foci of chondroitin sulphate staining prefigure emergence of the semicircular canal projections in wild-type ears

Versican genes, which code for the core proteins of chondroitin sulphate proteoglycans (CSPGs), are expressed in a dynamic pattern in the zebrafish ear, with maximum mRNA expression levels during projection outgrowth (Geng et al., 2013). To examine the distribution of chondroitin sulphate (CS) in the ear, we stained fixed samples with an antibody to CS. In phenotypically wild-type embryos (Section 2), anti-CS antibody staining revealed reproducible, small (10–20 µm diameter) foci that prefigure the emergence (evagination) of the lateral, anterior, posterior and ventral semicircular canal projections (Figure 1; Supplementary Movie S1; additional examples at different stages in Figure 4; Supplementary Figure S1A). For the anterior projection, staining was present at 40 hpf on the apical (lumenal) side of the epithelium (Figures 1A–C; see also Figures 4A, A’), with foci of staining present at the base of the lateral, anterior and posterior projections at 45–50 hpf (Figure 1E). For the ventral projection at 48–50 hpf, staining was present in a discrete patch or streak on the basal side of the epithelium, beneath a small group of cells that were about to evaginate to form the projection (Figure 1E; Supplementary Movie S1). By contrast, CS staining was absent from cells invaginating to form the endolymphatic duct, a short tube that extends from dorsal otic epithelium (Figures 1D,F). At later stages, after fusion of epithelial projections and bulges to form pillars, there was very little CS staining evident in the cores of the pillars or lateral projection in wild-type ears (Supplementary Movie S3; see also Figures 4E,G). Positive staining for CS also marked the otolithic membrane overlying sensory hair cells of the utricular macula (asterisks, Figure 1E, Supplementary Figure S1A–A”, Supplementary Movie S3).

### 3.2 The ears of *ext2* mutants, which have reduced heparan sulphate biosynthesis, have epithelial projection and otolith defects.

To test the role of heparan sulphate (HS) biosynthesis in otic morphogenesis, we imaged the developing ear in live zebrafish *ext2* (*dak* ^*tw25e*^) homozygous mutant embryos, at developmental stages when the otic epithelium is undergoing morphogenetic deformation to form the semicircular canal system (2–5 days post fertilisation, dpf). Otic development normally progresses by the formation of epithelial projections and bulges that fuse to form pillars of tissue spanning the otic lumen (Waterman and Bell, 1984; Haddon and Lewis, 1996). At 48 h post fertilisation (hpf), the *ext2* mutant otic vesicle was rounder in shape than that of phenotypically wild-type sibling embryos (Figures 2, 4). The lateral projection formed an anterior bulge, but formation of the posterior and ventral bulges was delayed or absent (Figures 2A,F’). By 72 hpf, epithelial projections in the wild-type ear had fused to form three pillars in all cases (*N* = 16 embryos; *n* = 32 ears; Figure 2C). By contrast, in *ext2* mutants at 72 hpf, the anterior projection had fused in some ears (*N =* 4 embryos; *n* = 5/8 ears), but the posterior projection had only fused in 1/8 mutant ears examined, and the ventral projection remained very small in all ears examined (*n =* 8/8). Unfused projections were also present in the ear at 4 and 5 days post fertilisation (dpf) (Figures 2D’,F’,H’), and the ears, although smaller than normal, became swollen, visible in a dorsal view (Figure 2F,F’; further examples in Figure 6). Some mutant ears also had abnormal out-pocketings of the epithelium, not present in the wild type (black arrowheads, Figure 2D,H’).

Measurements of ear size at 72 hpf indicated that the *ext2* mutant ears were narrower in the anteroposterior dimension in a lateral view (“ear width”) than in the wild type, with more variation in size and shape (Figure 2I; Supplementary Figure S2). Ear height in *ext2* mutants was also more variable, but the mean value was not significantly different from the wild type (Figure 2I; Supplementary Figure S2). Two otoliths were present in the *ext2* mutant ear, but were smaller than normal at 72 hpf (Figure 2J). However, the otoliths stain strongly with an antibody to keratan sulphate, as in the wild type (Figures 2K,L; Supplementary Figure S1B–B”). Phalloidin staining confirmed that all five sensory patches (two maculae and three cristae) were present in the *ext2* mutant ear at 3–5 dpf, and contained differentiated hair cells (Figure 2K,L; additional examples in Figure 4).

The *ext2* gene is expressed maternally and ubiquitously up to 36 hpf (Lee et al., 2004); transcript levels were found to be reduced in *ext2* mutants as measured by qPCR at 5 dpf (Wiweger et al., 2014). We used *in situ* hybridisation to examine the spatial expression pattern of *ext2* beyond 36 hpf (Supplementary Figure S3). Expression of *ext2* was regionalised at 50 and 72 hpf, with highest levels in the brain and retina, spinal cord, pectoral fin and pharyngeal region, whereas expression in the somites, lens and heart was weaker or undetectable. Expression was present in both the ear and lateral line neuromasts. mRNA for *ext2* was still expressed in *ext2* mutants, but levels appeared slightly reduced throughout, including in the ear at 72 hpf (Supplementary Figure S3).

### 3.3 Expression of *vcanb* and *chsy1* mRNA persists abnormally in the unfused epithelial projections of the *ext2* mutant ear

**FIGURE 3:**
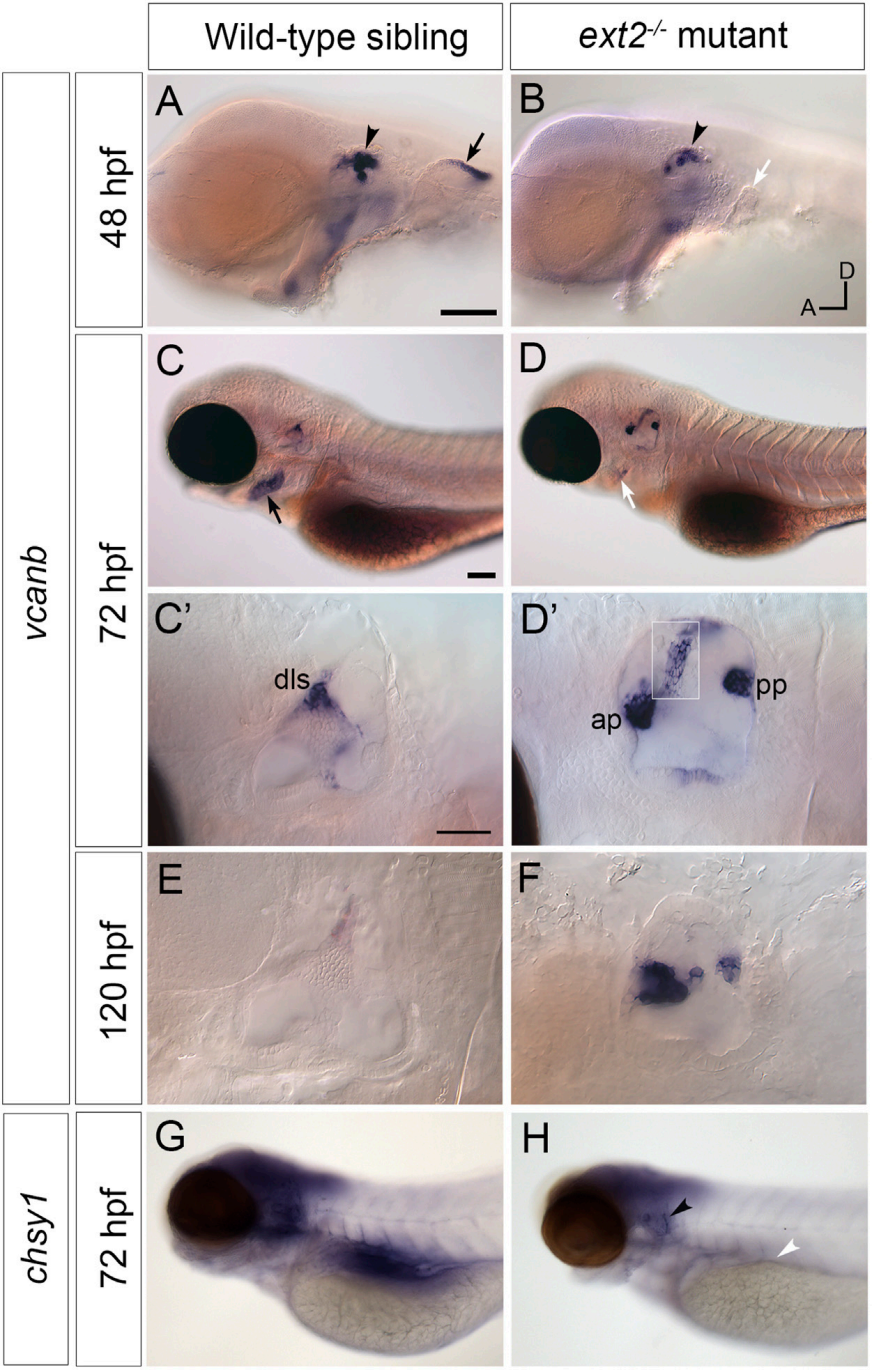
Expression of *vcanb* and *chsy1* in the *ext2* mutant ear. **(A–H)** *In situ* hybridisation to *vcanb* **(A–F)** and *chsy1* **(G,H)** mRNA in phenotypically wild-type sibling and *ext2* mutant embryos. **(A)** Expression of *vcanb* is present in the ear (black arrowhead) and pectoral fin (black arrow) in sibling embryos. **(B)** Only a rudimentary pectoral fin bud is present in the *ext2* mutant, and the associated *vcanb* staining is missing (white arrow). **(C,D)** At 72 hpf, *vcanb* expression is strongly reduced in the periderm of the second arch (future operculum) in *ext2* mutants [black arrowhead in **(C)**, white arrowhead in **(D)**]. **(C’,D’)** Enlargements of the ears shown in **(C,D)**, respectively. Expression of *vcanb* is now down-regulated in the pillars of the wild-type ear, remaining only in the dorsolateral septum (dls). In the *ext2* mutant, expression persists abnormally in the unfused anterior and posterior projections [ap, pp, **(D’)**] and in an abnormal pillar-like structure (boxed insert of same ear taken at a different focal plane). **(E,F)** *vcanb* expression persists abnormally in the unfused projections in mutant ears until at least 120 hpf. **(G,H)** Overall levels of *chsy1* expression are slightly reduced in *ext2* mutants **(H)**; expression is missing in the viscera (white arrowhead), but persists in unfused epithelial projections in the ear (black arrowhead). All panels are lateral views with anterior to the left, dorsal to the top (orientation shown in **(B)**: A, anterior; D, dorsal). Scale bars: in **(A)**, 100 μm (also applies to **(B)**); in **(C)**, 100 μm [also applies to **(D,G,H)**]; in **(C)**, 50 μm [also applies to **(D’)**].

We next tested the expression of *versican b* (*vcanb*) and *chondroitin synthase 1* (*chsy1*) mRNA in the wild-type and *ext2* mutant ear. Expression of *vcanb* in the wild-type ear is very similar to that of *vcana* (Geng et al., 2013), with high levels of expression in the epithelial projections as they evaginate and grow out into the lumen of the ear, becoming rapidly down-regulated after fusion to form a pillar (Figures 3A,C,E). Expression remains in the dorsolateral septum of the wild-type ear at 72 hpf (Figure 3C,C’), more weakly at 5 dpf (Figure 3E). Expression in the *ext2* mutants showed some striking differences to the wild-type pattern. At 48 hpf, otic *vcanb* mRNA expression levels were lower in all *ext2* mutants tested (26/26) (Figure 3A’). However, by 72 hpf, *vcanb* expression in the unfused epithelial projections was abnormally high, and persisted until at least 5 dpf (Figure 3D,D’,F). Mutants can easily be distinguished by their lack of pectoral fins, normally a strong site of *vcanb* expression at 50 hpf (Figure 3A,A’). Expression of *vcanb* in the periderm of the second arch (epidermis of the developing operculum) was also considerably reduced in *ext2* mutants (Figure 3,B’). The *chsy1* transcript, known to be expressed in the outgrowing projections of the wild-type ear (Li et al., 2010), was also mildly up-regulated in the unfused projections of the *ext2* mutant ear at 72 hpf, although overall levels in the brain of *ext2* mutants were slightly lower than in the wild type (Figures 3G,H).

### 3.4 The pattern of chondroitin sulphate staining is altered, but hyaluronan is still present, in the *ext2* mutant ear

**FIGURE 4:**
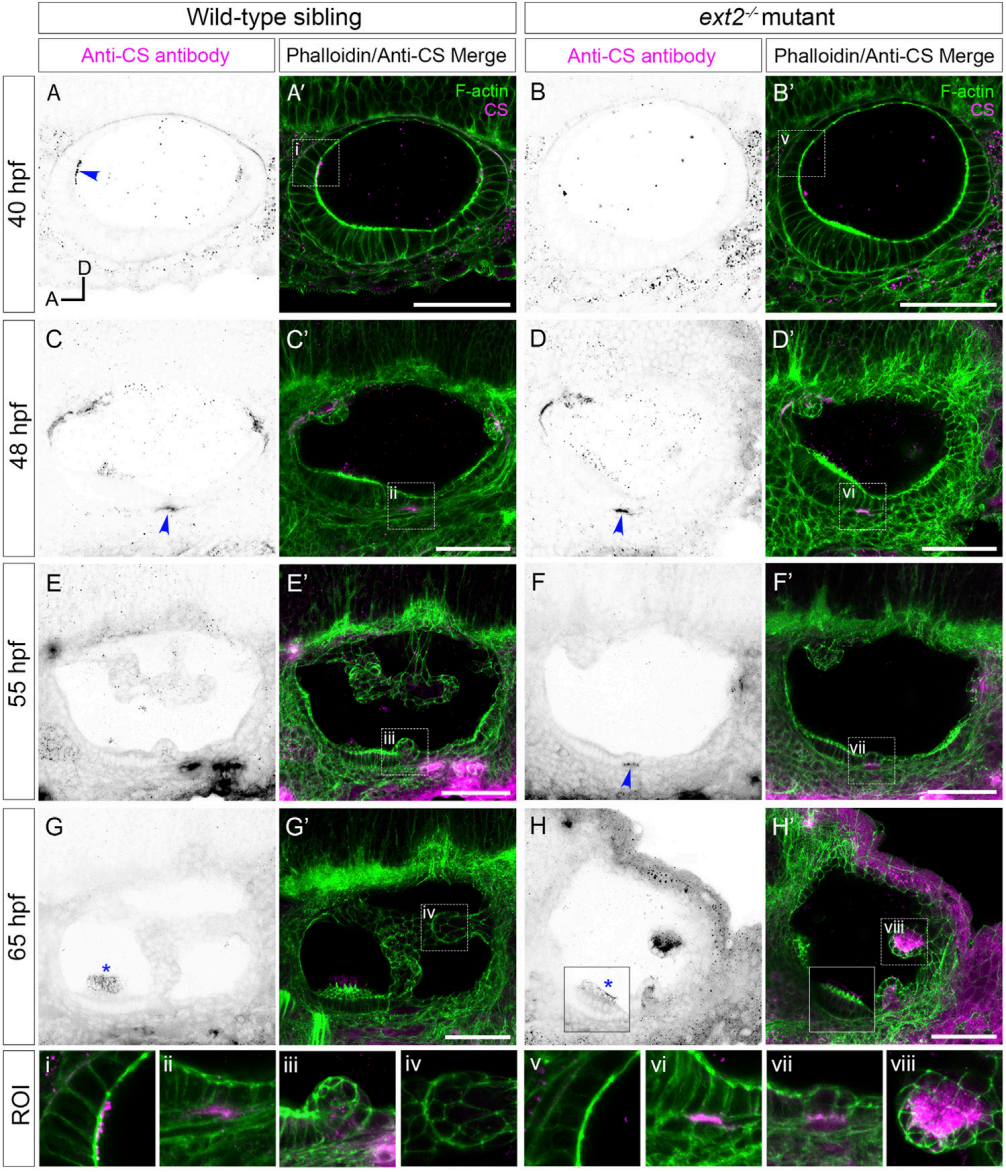
Staining for chondroitin sulphate in the *ext2* mutant ear is delayed at early stages, but accumulates abnormally in unfused epithelial projections by 65 hpf. **(A–H’)** Confocal images of Alexa-phalloidin (green) and anti-CS antibody (magenta) whole-mount stains of phenotypically wild-type sibling and *ext2* mutant ears (Section 2). Blue arrowheads mark foci of chondroitin sulphate (CS) staining associated with the emergence (evagination) of epithelial projections from the otic epithelium; blue asterisks mark staining in the otolithic membrane overlying hair cells of the anterior (utricular) macula. Boxed areas highlight regions of interest (ROI), enlarged in the bottom row of panels: **(i, v)**, apical focus of CS staining at 40 hpf, absent in the *ext2* mutant, prefiguring site of emergence of the anterior projection in the wild-type ear; **(ii, vi)**, foci of CS staining associated with the ventral projection at 48 hpf; **(iii, vii)**, CS staining in the emerging ventral projection, delayed in the *ext2* mutant; **(iv, viii)** no detectable staining in the wild-type posterior projection **(iv)** contrasts with strong staining in the unfused posterior projection in the *ext2* mutant **(viii)**. The boxed area in the lower left of **(H, H’)** is taken from a different focal plane in the stack to show the anterior (utricular) macula and otolithic membrane. All images are maximum intensity projections (MIPs) of between 3 and 10 selected *z*-slices, with the exception of the images at 40 hpf, which are single *z*-slices. All images are lateral views with anterior to left and dorsal to top (orientation shown in **(A)**: A, anterior; D, dorsal). Scale bars, 50 µm. Abbreviation: CS, chondroitin sulphate.

Overall levels of chondroitin sulphate (CS) were previously found to be normal in zebrafish *ext2* mutants at 6 dpf by reverse-phase ion-pair high-performance liquid chromatography (Holmborn et al., 2012). We used antibody staining to examine the spatial pattern of chondroitin sulphate (CS) in *ext2* mutant ears (Figure 4; Supplementary Movies S2,S4). The apical staining seen in phenotypically wild-type sibling ears at 40–45 hpf was not present in the mutant, even at later stages (Figure 4A–B’). Basal staining prefiguring projection outgrowth was delayed relative to that in the wild type, but otherwise appeared normal (Figures 4C–F’). At 48 hpf, a discrete patch of CS staining was found directly posterior to the anterior (utricular) macula in both wild-type and mutant embryos, marking the site of emergence of the ventral projection. In the example shown in Figure 4F’, staining was specifically localised to the basal side of the cells that have just started to evaginate and move into the otic lumen. By 65 hpf, little CS signal remained in the acellular cores of the semicircular canal pillars in the wild-type ear (Figures 4G,G’; Supplementary Movie S3). By contrast, CS staining in the *ext2* mutant ear, like expression of *vcanb*, persisted abnormally and strongly in the unfused epithelial projections (Figures 4H, H’, Supplementary Movie S4). Staining for CS indicated that an otolithic membrane was present in the *ext2* mutant, although staining at 65 hpf was reduced compared with that in the wild type, possibly indicating a thinner membrane (Figures 4G–H’, blue asterisks; Supplementary Movies S3,S4).

To test whether hyaluronan (HA) is produced in the *ext2* mutant ear, we stained embryos for the expression of *ugdh*, a gene required for the production of HA, and with a biotinylated hyaluronic acid binding protein (HABP). Expression of *ugdh* was present, but reduced in levels, throughout the head of *ext2* mutant embryos, including in the ear (Supplementary Figure S4). Staining with HABP indicated that HA was still present in the cores of the unfused epithelial projections of the *ext2* mutant ear (Supplementary Figure S5).

### 3.5 Otoliths in the *ext2* mutant ear are not tethered correctly to the saccular macula

**FIGURE 5:**
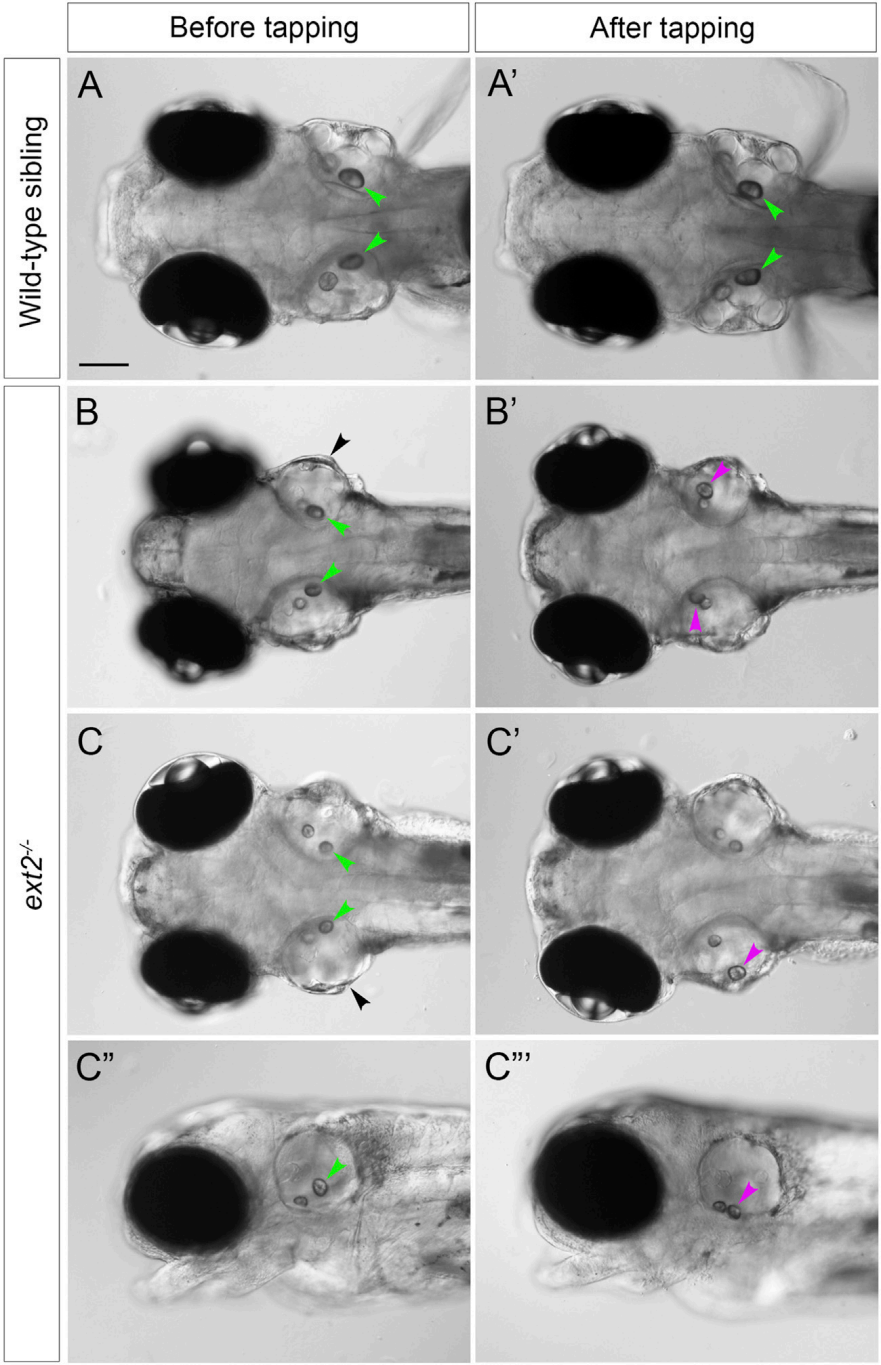
Saccular otoliths are not tethered correctly in the homozygous *ext2* mutant ear at 5 dpf. **(A–C”’)** Live DIC images of embryos at 5 dpf, before (left-hand column) and after (right-hand column) tapping. In phenotypically wild-type sibling embryos **(A,A’)**, saccular otoliths (green arrowheads) remain in place after tapping (in this example, one of the utricular otoliths in **(A’)** has become slightly displaced). In *ext2* mutant embryos **(B–D’)**, the saccular otolith in one or both ears becomes displaced (magenta arrowheads show new position after tapping). **(A–C’)** are dorsal views with anterior to the left; **(C”,C”’)** are lateral views of the embryo shown in **(C,C’)**. Note also the swollen ear morphology in the *ext2* mutants [**(B,C)**, black arrowheads]. In all panels, anterior is to the left. Scale bar in **(A)**, 100 μm (applies to all panels).

The posterior otolith (sagitta) frequently appeared misplaced in *ext2* mutants between 72 hpf and 5 dpf (Figures 2G,H), indicating defective tethering to its cognate sensory patch (the saccular macula). To investigate further, we performed a simple experiment to test whether the otolith could become dislodged after tapping the slide on which the fish was mounted (see Methods). Images of the ears were taken before and after tapping (Figure 5). In wild-type ears, all saccular otoliths (sagittae) remained in place after tapping (*N =* 8 embryos, *n* = 16 otoliths), whereas in 7 out of 8 *ext2* mutant embryos, one or both sagittae were displaced (Figure 5). Unlike the sagitta, the anterior otolith (lapillus) did not appear misplaced in any of the mutant embryos after tapping (*N* = 8 mutant embryos).

### 3.6 Genes involved in otolith formation and tethering show reduced expression in the *ext2* mutant ear

**FIGURE 6:**
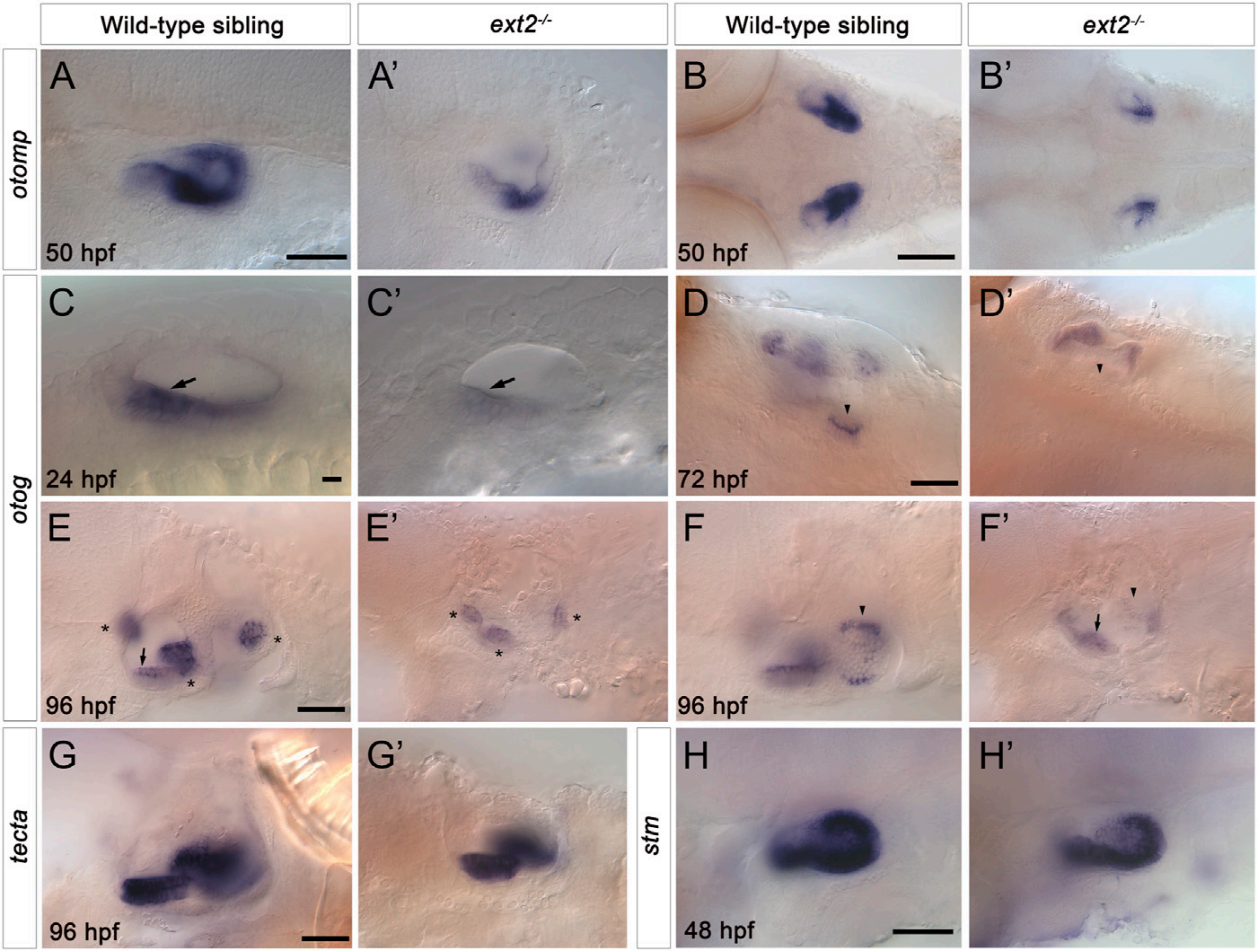
Expression of *otomp*, *otog* and *stm* is reduced in the saccular macula of the *ext2* ^*−/−*^ mutant ear. **(A–H’)** *In situ* hybridisation to otolith marker genes in the ears of phenotypically wild-type sibling and *ext2* mutant embryos. **(A–B’)** Levels of *otomp* expression in the *ext2* mutant ear **(A’,B’)** at 50 hpf were reduced compared with the wild type **(A,B)**. **(C–F’)** Expression of *otog* at 24 hpf **(C,C’)**, 72 hpf **(D,D’)**, and 96 hpf **(E-F’)** was significantly reduced in the *ext2* mutant ear. The images in **(F,F’)** show a more medial focal plane of the same ears depicted in **(E,E’)**, respectively. **(G,G’)** Expression of *tecta* in the *ext2* mutant ear was unaltered at 96 hpf. **(H,H’)** Expression of *stm* expression at 48 hpf was reduced in the posterior macula (arrowhead) of the *ext2* mutant ear. Arrowheads mark the posterior (saccular) macula; arrows mark the anterior (utricular) macula; asterisks mark the cristae. All images are lateral views with anterior to the left, apart from **(B,B’,D,D’)** (dorsal views with anterior to the left). In all panels, anterior is to the left. Scale bars: in **(A)**, 50 μm for **(A’)**; in **(B)**, 100 μm for **(B’)**; in **(C)**, 10 μm for **(C’)**; in **(D)**, 50 μm for **(D’)**; in **(E)**, 50 μm for **(E’–F’)**; in **(G)**, 50 μm for **(G’)**; in **(H)**, 50 μm for **(H’)**.

To test whether the otolith size and tethering defects reflect changes in the expression of genes coding for otolith matrix or otolithic membrane components, we examined the expression of selected markers using *in situ* hybridisation (Figure 6). Expression of *otolith matrix protein* (*otomp*) mRNA was significantly reduced in both the anterior and the posterior maculae in *ext2* ^*−/−*^ mutants (Figures 6A–B’). Expression of *starmaker* (*stm*) was present in both maculae in *ext2* mutants, with levels slightly reduced in the posterior (saccular) macula (Figures 6C,C’). We also analysed the expression of mRNA for *otogelin* (*otog*) and *alpha-tectorin* (*tecta*). Expression of *otog* was significantly reduced in the anterior (utricular) macula of *ext2* mutants at 24 hpf, and was absent from the posterior (saccular) macula from 72 to 120 hpf (Figures 6I–F’). Expression of *otog* in the three sensory cristae was also reduced in *ext2* mutants (Figures 6E,E’). However, expression of *tecta* mRNA appeared normal in the *ext2* mutant ear (Figures 6I–J’).

## 4 Discussion

### 4.1 Presence of chondroitin sulphate marks initial sites of evagination of the otic epithelium

An unsolved question in zebrafish semicircular canal duct morphogenesis is what establishes the site of evagination of each of the epithelial projections, where a small number of cells start to move towards the lumen of the ear, involving a localised inversion of curvature of the epithelial sheet. To begin to answer this, our results identify the earliest signs of projection outgrowth: exquisitely localised patches of CS staining marking the sites where the epithelium will deform, present before any obvious morphological changes. These observations corroborate the finding that *chsy1*, which codes for the enzyme Chondroitin synthase 1, is expressed in the epithelial projections of the ear, and is required for their normal morphogenesis (Li et al., 2010). A similar transient and spatially restricted pattern of CS staining has been observed in the developing zebrafish heart, where it is required for normal atrioventricular canal formation (Peal et al., 2009). In the ear, CS staining was absent from cells forming the endolymphatic duct (ED), a tube that invaginates from dorsal otic epithelium. Unlike the evaginating epithelial projections, formation of the ED does not require any inversion of epithelial curvature. ED cells show apical constriction (Swinburne et al., 2018), and differ significantly in shape from those of the epithelial projections (Mendonca et al., 2021). Thus, the presence or absence of CS distinguishes between deformations of the epithelium with opposite orientations in the zebrafish ear; it will be interesting to see whether this is a general principle.

### 4.2 Growth of the epithelial projections in the zebrafish ear requires both hyaluronan and proteoglycans: proposal for a hyaluronan-proteoglycan-driven mechanism

Numerous lines of evidence support a model in which the synthesis of the non-sulphated GAG hyaluronan (HA), a giant linear polysaccharide of several million Daltons (reviewed in Toole, 2001), acts to propel the epithelial projections into the lumen of the anamniote inner ear. Local enzymatic digestion of HA, or a systemic block to HA biosynthesis, results in rudimentary epithelial projections that fail to meet and fuse (Haddon and Lewis, 1991; Busch-Nentwich et al., 2004; Geng et al., 2013; Munjal et al., 2021). However, the similar otic phenotypes in zebrafish morphants for *chsy1* (Li et al., 2010) and mutants for *ext2* (this work) demonstrate that synthesis of chondroitin sulphate (CS) and heparan sulphate (HS), respectively, are also critical for normal epithelial projection outgrowth. As HA is still present in *ext2* mutant ears, this argues that HA alone is not sufficient for normal epithelial projection outgrowth, and that CSPGs and HSPGs are likely to play a role. In addition, ECM proteins expressed in the projections, such as the Type II collagens (Geng et al., 2013), might also contribute to projection outgrowth.

One of the remarkable aspects of semicircular canal morphogenesis in the zebrafish or *Xenopus* ear is the finger-like nature of the evaginating epithelial protrusions or projections, differing markedly from the walls of the flattened out-pocketings or pouches of the otic epithelium that form in the amniote ear (reviewed in Alsina and Whitfield, 2017). In the mouse, reciprocal signalling between otic epithelium and periotic mesenchyme, resulting in mesenchymal cell proliferation, is thought to be required to drive the epithelial walls of the pouches together to meet at a fusion plate (Salminen et al., 2000; Pirvola et al., 2004). By contrast, the epithelial projections in zebrafish are not underlain by mesenchyme as they grow into the otic lumen; instead, each projection has an acellular (matrix-filled) core. With only three or 4 cells at their tip (Waterman and Bell, 1984), these projections represent a highly anisotropic mode of growth. Haddon and Lewis recognised that HA must not only be synthesised locally but also be held in place in order to generate localised outgrowth (Haddon and Lewis, 1991). Munjal and others have extended this model, proposing that differential tissue stiffness, mediated by tensioned cell tethers, in combination with isotropic pressure from HA, acts to shape the projections (Munjal et al., 2021).

It is also possible that aggregation of proteoglycans and HA could generate a local expansion of the ECM beneath cells of the projection. HSPGs and CSPGs bind to HA via hyaluronan-proteoglycan link proteins (Haplns), generating large charged aggregates (reviewed in Toole, 2001). The Hapln genes *hapln1a* and *hapln3* are expressed in the epithelial projections of the zebrafish ear (Geng et al., 2013). Addition of proteoglycans is sufficient to cause the ‘drastic swelling’ of HA films *in vitro*, where linear HA chains, normally present in a random coil configuration, become stretched on incorporation of relatively small quantities of proteoglycan (Attili and Richter, 2013; Chang et al., 2016; reviewed in Richter et al., 2018). There are interesting parallels with the Hapln1a-dependent left-sided expansion of the cardiac ECM that occurs in the developing zebrafish heart prior to looping (Derrick et al., 2021). In the ear, anisotropy of the matrix in the projection cores, mediated by the stretching of HA chains driven by localised incorporation of proteoglycan, or alignment of other fibrillar components such as collagen, could help to direct projection outgrowth: this idea remains to be tested. The sulphation of HSPGs and CSPGs also confers a strong negative charge, important for hydration of the matrix. Diversity in the proteoglycan sulphation pattern, mediated by the enzymes coded for by *sulf* genes, some of which are expressed in the zebrafish ear (Gorsi et al., 2010; Meyers et al., 2013; Habicher et al., 2015), might provide further local heterogeneity in the mechanical properties of the otic ECM.

### 4.3 Implications for cell signalling in the developing ear

As in other species, zebrafish proteoglycans play an important role in the correct function of various signalling pathways, including Fgf, BMP and Wnt (see, for example, Norton et al., 2005; Li et al., 2010; Fischer et al., 2011; Meyers et al., 2013; Venero Galanternik et al., 2015). Although this was not the focus of our study, it is likely that disrupted signalling also contributes to the morphogenetic and patterning defects in the *ext2* mutant ear. Expression of *fgf8a* in the utricular macula appears relatively normal in the ears of *ext2* ^*tw25e*^ mutants at 48 hpf, but the Fgf target gene *etv5b* is downregulated in various tissues, including the otic vesicle (Fischer et al., 2011). By contrast, expression of the Fgf target gene *pea3* appeared unchanged in the ear in *ext2* mutants or by treatment with SU5402 alone, but was strongly downregulated in SU5402-treated *ext2* mutants (Fischer et al., 2011). A detailed comparison of otic phenotypes will be important to reveal any similarities between the ears of *ext2* mutants and those with disrupted Fgf, BMP or Wnt signalling.

### 4.4 Development and tethering of otoliths in the *ext2* mutant ear

In addition to semicircular canal defects, the *ext2* mutant ear has otolith size and tethering abnormalities. Morpholino-mediated knockdown of *otomp* has been linked to slowed otolith growth (Murayama et al., 2005), and *otomp* expression is reduced in *ext2* mutant ears. Although otoliths were small in the *ext2* mutant, they stained for keratan sulphate (KS) as in the wild type. Keratan sulphate proteoglycans (KSPGs) are well-known constituents of amniote otoconia (reviewed in Lundberg et al., 2015). In the chicken, alpha-Tectorin, a major glycoprotein constitutent of the tectorial membrane, has characteristics of a “light” KSPG (Killick and Richardson, 1997), and KS on alpha-Tectorin might account for the otolithic membrane KS staining seen in the zebrafish. In zebrafish, Otogelin and alpha-Tectorin, respectively, establish and maintain otolith adhesion to the macula during otolith growth and biomineralisation (Stooke-Vaughan et al., 2015). A loss of adhesion of the sagitta (saccular otolith) in *ext2* mutants might be attributed to the lack of *otogelin* expression in the saccular macula. However, the early *ext2* mutant phenotype does not resemble that of the *otogelin* mutant, where only one otolith forms in each mutant ear (Stooke-Vaughan et al., 2015). Instead, the loss of saccular otolith tethering in the *ext2* mutant is more reminiscent of the mutant phenotype for *tecta*, where the sagittae can become dislodged between 3 dpf and 5 dpf, but the lapilli (utricular otoliths) are unaffected (Stooke-Vaughan et al., 2015). *Tecta* mRNA is expressed normally in the *ext2* mutant ear, and so it is not possible to attribute the otolith tethering defect to a loss of alpha-Tectorin. However, the *ext2* mutant phenotype suggests that one or more HSPGs are required for formation of a normal otolithic membrane and for normal otolith tethering. Given the capacity of HS to interact with a large number of proteins (Vallet et al., 2022), HS-protein binding is likely to contribute to otolith tethering in the zebrafish ear.

## 5 Conclusion

In summary, our data show that the temporally- and spatially-restricted presence of CS marks the sites of epithelial projection outgrowth in the zebrafish ear. In addition, otic defects in the *ext2* mutant demonstrate that HS biosynthesis is essential for the evagination and growth of the epithelial projections, and for otolith tethering. These findings suggest that CSPGs and HSPGs, as well as HA, play important roles in semicircular canal duct morphogenesis, and are likely to impact both the mechanical and signalling properties of the otic epithelium.

## Data Availability

The original contributions presented in the study are included in the article/Supplementary Material; further inquiries can be directed to the corresponding author.

## References

[B1] Aleström P. D’Angelo L. Midtlyng P. J. Schorderet D. F. Schulte-Merker S. Sohm F. (2020). Zebrafish: Housing and husbandry recommendations. Lab. Anim. 54, 213–224. 10.1177/0023677219869037 31510859 PMC7301644

[B2] Alsina B. Whitfield T. T. (2017). Sculpting the labyrinth: Morphogenesis of the developing inner ear. Semin. Cell Dev. Biol. 65, 47–59. 10.1016/j.semcdb.2016.09.015 27686400

[B3] Attili S. Richter R. P. (2013). Self-assembly and elasticity of hierarchical proteoglycan-hyaluronan brushes. Soft Matter 9, 10473. 10.1039/c3sm51213d

[B4] Bülow H. E. Hobert O. (2006). The molecular diversity of glycosaminoglycans shapes animal development. Annu. Rev. Cell Dev. Biol. 22, 375–407. 10.1146/annurev.cellbio.22.010605.093433 16805665

[B5] Busch-Nentwich E. Söllner C. Roehl H. Nicolson T. (2004). The deafness gene dfna5 is crucial for ugdh expression and HA production in the developing ear in zebrafish. Development 131, 943–951. 10.1242/dev.00961 14736743

[B6] Chang P. S. McLane L. T. Fogg R. Scrimgeour J. Temenoff J. S. Granqvist A. (2016). Cell surface access is modulated by tethered bottlebrush proteoglycans. Biophys. J. 110, 2739–2750. 10.1016/j.bpj.2016.05.027 27332132 PMC4919654

[B7] Clément A. Wiweger M. von der Hardt S. Rusch M. A. Selleck S. B. Chien C.-B. (2008). Regulation of Zebrafish Skeletogenesis by ext2/dackel and papst1/pinscher. PLoS Genet. 4, e1000136. 10.1371/journal.pgen.1000136 18654627 PMC2453328

[B8] Derrick C. J. Sánchez-Posada J. Hussein F. Tessadori F. Pollitt E. J. G. Savage A. M. (2021). Asymmetric Hapln1a drives regionalized cardiac ECM expansion and promotes heart morphogenesis in zebrafish development. Cardiovasc. Res., 1–15. 10.1101/838128 PMC8752364 33616638

[B9] Filipek-Górniok B. Holmborn K. Haitina T. Habicher J. Oliveira M. B. Hellgren C. (2013). Expression of chondroitin/dermatan sulfate glycosyltransferases during early zebrafish development. Dev. Dyn. 242, 964–975. 10.1002/dvdy.23981 23703795

[B10] Fischer S. Filipek-Gorniok B. Ledin J. (2011). Zebrafish Ext2 is necessary for Fgf and Wnt signaling, but not for Hh signaling. 10.1186/1471-213X-11-53 PMC3183004 21892940

[B11] Geng F.-S. Abbas L. Baxendale S. Holdsworth C. J. Swanson A. G. Slanchev K. (2013). Semicircular canal morphogenesis in the zebrafish inner ear requires the function of *gpr126* (*lauscher*), an adhesion class G protein-coupled receptor gene. Development 140, 4362–4374. 10.1242/dev.098061 24067352 PMC4007713

[B12] Gorsi B. Whelan S. Stringer S. E. (2010). Dynamic expression patterns of 6-O endosulfatases during zebrafish development suggest a subfunctionalisation event for sulf2, 3312–3323. 10.1002/dvdy.22456 20981828

[B13] Habicher J. Haitina T. Eriksson I. Holmborn K. Dierker T. Ahlberg P. E. (2015). Chondroitin/dermatan sulfate modification enzymes in zebrafish development. PLoS One 10, e0121957. 10.1371/journal.pone.0121957 25793894 PMC4368567

[B14] Häcker U. Lin X. Perrimon N. Hacker U. (1997). The Drosophila sugarless gene modulates Wingless signaling and encodes an enzyme involved in polysaccharide biosynthesis. Development 124, 3565–3573. 10.1242/dev.124.18.3565 9342049

[B15] Haddon C. Lewis J. (1996). Early ear development in the embryo of the zebrafish, *Danio rerio* . J. Comp. Neurol. 365, 113–128. 10.1002/(SICI)1096-9861(19960129)365:1<113::AID-CNE9>3.0.CO;2-6 8821445

[B16] Haddon C. M. Lewis J. H. (1991). Hyaluronan as a propellant for epithelial movement: The development of semicircular canals in the inner ear of *Xenopus* . Development 112, 541–550. 10.1242/dev.112.2.541 1794322

[B17] Haffter P. Granato M. Brand M. Mullins M. C. Hammerschmidt M. Kane D. A. (1996). The identification of genes with unique and essential functions in the development of the zebrafish, *Danio rerio* . Development 123, 1–36. 10.1242/dev.123.1.1 9007226

[B18] Holmborn K. Habicher J. Kasza Z. Eriksson A. S. Filipek-Gorniok B. Gopal S. (2012). On the roles and regulation of chondroitin sulfate and heparan sulfate in zebrafish pharyngeal cartilage morphogenesis. J. Biol. Chem. 287, 33905–33916. 10.1074/jbc.M112.401646 22869369 PMC3460485

[B19] Kang J. S. Oohashi T. Kawakami Y. Bekku Y. Izpisúa Belmonte J. C. Ninomiya Y. (2004). Characterization of *dermacan*, a novel zebrafish lectican gene, expressed in dermal bones. Mech. Dev. 121, 301–312. 10.1016/j.mod.2004.01.007 15003632

[B20] Karlstrom R. O. Trowe T. Klostermann S. Baier H. Brand M. Crawford A. D. (1996). Zebrafish mutations affecting retinotectal axon pathfinding. Development 123, 427–438. 10.1242/dev.123.1.427 9007260

[B21] Killick R. Richardson G. P. (1997). Antibodies to the sulphated, high molecular mass mouse tectorin stain hair bundles and the olfactory mucus layer. Hear. Res. 103, 131–141. 10.1016/s0378-5955(96)00174-8 9007580

[B22] Kimmel C. B. Ballard W. W. Kimmel S. R. Ullmann B. Schilling T. F. (1995). Stages of embryonic development of the zebrafish. Dev. Dyn. 203, 253–310. 10.1002/aja.1002030302 8589427

[B23] Lee J. S. von der Hardt S. Rusch M. A. Stringer S. E. Stickney H. L. Talbot W. S. (2004). Axon sorting in the optic tract requires HSPG synthesis by *ext2* (*dackel*) and *extl3* (*boxer*). Neuron 44, 947–960. 10.1016/j.neuron.2004.11.029 15603738

[B24] Li Y. Laue K. Temtamy S. Aglan M. Kotan L. D. Yigit G. (2010). Temtamy preaxial brachydactyly syndrome is caused by loss-of-function mutations in chondroitin synthase 1, a potential target of BMP signaling. Am. J. Hum. Genet. 87, 757–767. 10.1016/j.ajhg.2010.10.003 21129728 PMC2997369

[B25] Lister J. A. Robertson C. P. Lepage T. Johnson S. L. Raible D. W. (1999). *Nacre* encodes a zebrafish microphthalmia-related protein that regulates neural-crest-derived pigment cell fate. Development 126, 3757–3767. 10.1242/dev.126.17.3757 10433906

[B26] Lundberg Y. W. Xu Y. Thiessen K. D. Kramer K. L. (2015). Mechanisms of otoconia and otolith development, 239–253. 10.1002/dvdy.24195 PMC4482761 25255879

[B27] Mendonca T. Jones A. A. Pozo J. M. Baxendale S. Whitfield T. T. Frangi A. F. (2021). Origami: Single-cell 3D shape dynamics oriented along the apico-basal axis of folding epithelia from fluorescence microscopy data. PLoS Comput. Biol. 17, e1009063. 10.1371/journal.pcbi.1009063 34723957 PMC8584784

[B28] Meyers J. R. Planamento J. Ebrom P. Krulewitz N. Wade E. Pownall E. (2013). Sulf1 modulates BMP signaling and is required for somite morphogenesis and development of the horizontal myoseptum. Dev. Biol. 378, 107–121. 10.1016/j.ydbio.2013.04.002 23583585

[B29] Munjal A. Hannezo E. Tsai T. Y. Mitchison T. J. Megason S. G. (2021). Extracellular hyaluronate pressure shaped by cellular tethers drives tissue morphogenesis. Cell 184, 6313–6325.e18. 10.1016/j.cell.2021.11.025.e18 34942099 PMC8722442

[B30] Murayama E. Herbomel P. Kawakami A. Takeda H. Nagasawa H. (2005). Otolith matrix proteins OMP-1 and Otolin-1 are necessary for normal otolith growth and their correct anchoring onto the sensory maculae. Mech. Dev. 122, 791–803. 10.1016/j.mod.2005.03.002 15905077

[B31] Neuhauss S. C. F. Solnica-Krezel L. Schier A. F. Zwartkruis F. Stemple D. L. Malicki J. (1996). Mutations affecting craniofacial development in zebrafish. Development 123, 357–367. 10.1242/dev.123.1.357 9007255

[B32] Norton W. H. J. Ledin J. Grandel H. Neumann C. J. (2005). HSPG synthesis by zebrafish Ext2 and Extl3 is required for Fgf10 signalling during limb development. Development 8, 4963–4973. 10.1242/dev.02084 16221725

[B33] Peal D. S. Burns C. G. Macrae C. A. Milan D. (2009). Chondroitin sulfate expression is required for cardiac atrioventricular canal formation. Dev. Dyn. 238, 3103–3110. 10.1002/dvdy.22154 19890913 PMC2852642

[B34] Pirvola U. Zhang X. Mantela J. Ornitz D. M. Ylikoski J. (2004). Fgf9 signaling regulates inner ear morphogenesis through epithelial-mesenchymal interactions. Dev. Biol. 273, 350–360. 10.1016/j.ydbio.2004.06.010 15328018

[B35] Richter R. P. Baranova N. S. Day A. J. Kwok J. C. (2018). Glycosaminoglycans in extracellular matrix organisation: Are concepts from soft matter physics key to understanding the formation of perineuronal nets? Curr. Opin. Struct. Biol. 50, 65–74. 10.1016/j.sbi.2017.12.002 29275227

[B36] Salminen M. Meyer B. I. Bober E. Gruss P. (2000). Netrin 1 is required for semicircular canal formation in the mouse inner ear. Development 127, 13–22. 10.1242/dev.127.1.13 10654596

[B37] Schilling T. F. Piotrowski T. Grandel H. Brand M. Heisenberg C.-P. Jiang Y.-J. (1996). Jaw and branchial arch mutants in zebrafish I: Branchial arches. Development 123, 329–344. 10.1242/dev.123.1.329 9007253

[B38] Schindelin J. Arganda-Carreras I. Frise E. Kaynig V. Longair M. Pietzsch T. (2012). Fiji: An open-source platform for biological-image analysis. Nat. Methods 9, 676–682. 10.1038/nmeth.2019 22743772 PMC3855844

[B39] Söllner C. Burghammer M. Busch-Nentwich E. Berger J. Schwarz H. Riekel C. (2003). Control of crystal size and lattice formation by Starmaker in otolith biomineralization. Science 302, 282–286. 10.1126/science.1088443 14551434

[B40] Stooke-Vaughan G. A. Obholzer N. D. Baxendale S. Megason S. G. Whitfield T. T. (2015). Otolith tethering in the zebrafish otic vesicle requires Otogelin and alpha-Tectorin. Development 142, 1137–1145. 10.1242/dev.116632 25758224 PMC4360185

[B41] Swinburne I. A. Mosaliganti K. R. Upadhyayula S. Liu T. L. Hildebrand D. G. C. Tsai T. Y. C. (2018). Lamellar projections in the endolymphatic sac act as a relief valve to regulate inner ear pressure. Elife 7, e37131. 10.7554/eLife.37131 29916365 PMC6008045

[B42] Thisse C. Thisse B. (2008). High-resolution *in situ* hybridization to whole-mount zebrafish embryos. Nat. Protoc. 3, 59–69. 10.1038/nprot.2007.514 18193022

[B43] Toole B. P. (2001). Hyaluronan in morphogenesis. Semin. Cell Dev. Biol. 12, 79–87. 10.1006/scdb.2000.0244 11292373

[B44] Trowe T. Klostermann S. Baier H. Granato M. Crawford A. D. Grunewald B. (1996). Mutations disrupting the ordering and topographic mapping of axons in the retinotectal projection of the zebrafish, *Danio rerio* . Development 123, 439–450. 10.1242/dev.123.1.439 9007261

[B45] Vallet S. D. Berthollier C. Ricard-Blum S. (2022). The glycosaminoglycan interactome 2.0. Am J Physiol Cell Physiol. 322, C1271–C1278. 10.1152/ajpcell.00095.2022 35544698

[B46] van Eeden F. J. M. Granato M. Schach U. Brand M. Furutani-Seiki M. Haffter P. (1996). Genetic analysis of fin formation in the zebrafish , *Danio rerio* . Development 123, 255–262. 10.1242/dev.123.1.255 9007245

[B47] Venero Galanternik M. Kramer K. L. Piotrowski T. (2015). Heparan sulfate proteoglycans regulate fgf signaling and cell polarity during collective cell migration. Cell Rep. 10, 414–428. 10.1016/j.celrep.2014.12.043 25600875 PMC4531098

[B48] Walsh E. C. Stainier D. Y. R. (2001). UDP-glucose dehydrogenase required for cardiac valve formation in zebrafish. Science. 293, 1670–1673. 10.1126/science.293.5535.1670 11533493

[B49] Waterman R. E. Bell D. H. (1984). Epithelial fusion during early semicircular canal formation in the embryonic zebrafish, *Brachydanio rerio* . Anat. Rec. 210, 101–114. 10.1002/ar.1092100113 6486477

[B50] Westerfield M. (2007). The zebrafish book: A guide for the laboratory use of zebrafish (*Danio rerio*). Eugene: University of Oregon Press.

[B51] Whitfield T. T. Granato M. van Eeden F. J. M. Schach U. Brand M. Furutani-Seiki M. (1996). Mutations affecting development of the zebrafish inner ear and lateral line. Development 123, 241–254. 10.1242/dev.123.1.241 9007244

[B52] Wiweger M. I. De Andrea C. E. Scheepstra K. W. F. Zhao Z. Hogendoorn P. C. W. (2014). Possible effects of EXT2 on mesenchymal differentiation - lessons from the zebrafish. Orphanet J. Rare Dis. 9, 35. 10.1186/1750-1172-9-35 24628984 PMC4004154

[B53] Wiweger M. I. Zhao Z. van Merkesteyn R. J. Roehl H. H. Hogendoorn P. C. (2012). HSPG-deficient zebrafish uncovers dental aspect of multiple osteochondromas. PLoS One 7, e29734. 10.1371/journal.pone.0029734 22253766 PMC3256178

[B54] Zimmer B. M. Barycki J. J. Simpson M. A. (2021). Integration of sugar metabolism and proteoglycan synthesis by UDP-glucose dehydrogenase. J. Histochem. Cytochem. 69, 13–23. 10.1369/0022155420947500 32749901 PMC7780191

