# Supplementary figures and images for: Presence of chondroitin sulphate and requirement for heparan sulphate biosynthesis in the developing zebrafish inner ear
Source: Front Cell Dev Biol. 2022 Aug 26;10:959624. doi: 10.3389/fcell.2022.959624 (PMC9458858; doi:10.3389/fcell.2022.959624)

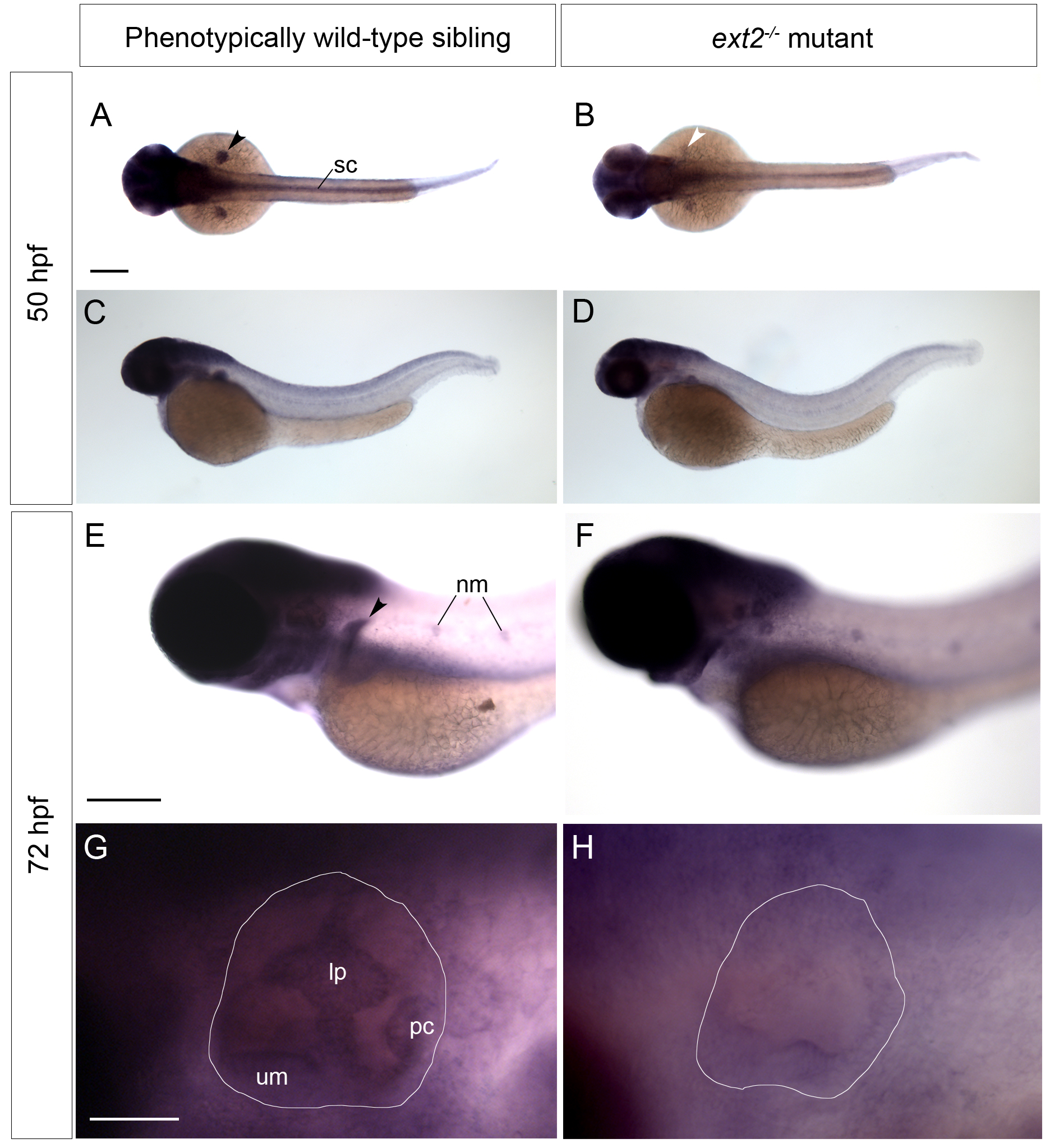

Supplement: Supplementary file 2 [file Image3.JPEG]

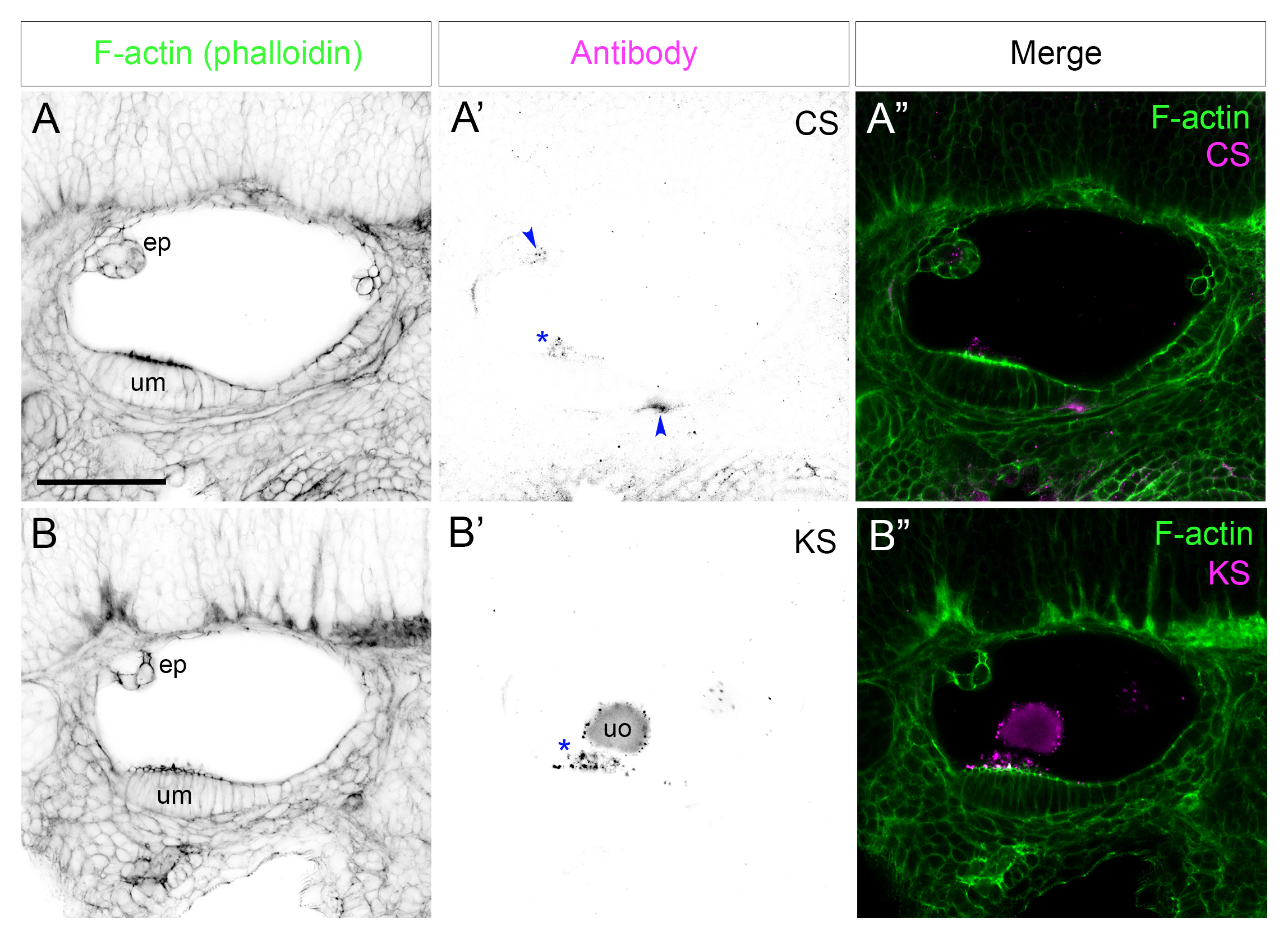

Supplement: Supplementary file 4 [file Image1.JPEG]

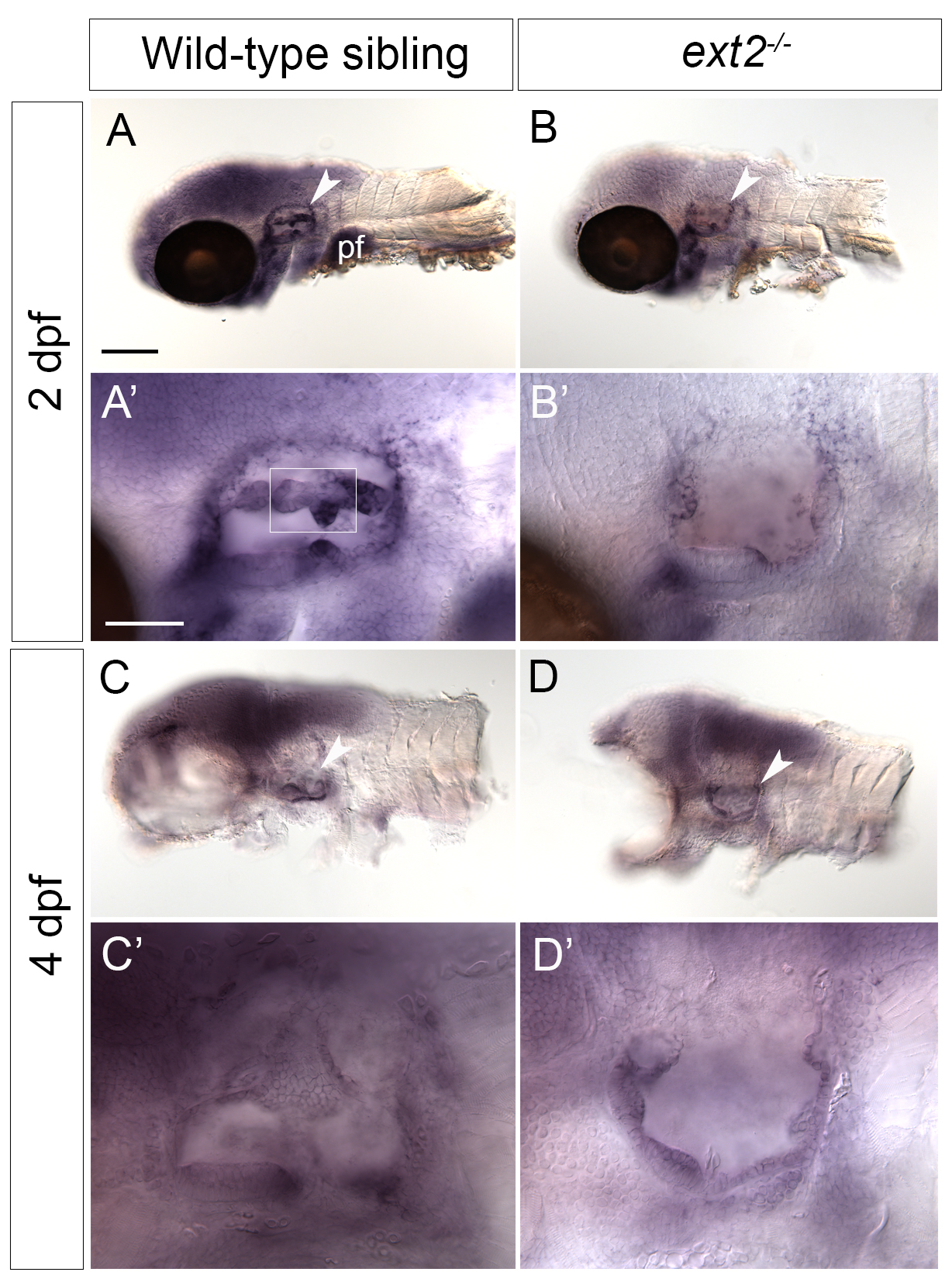

Supplement: Supplementary file 5 [file Image4.JPEG]

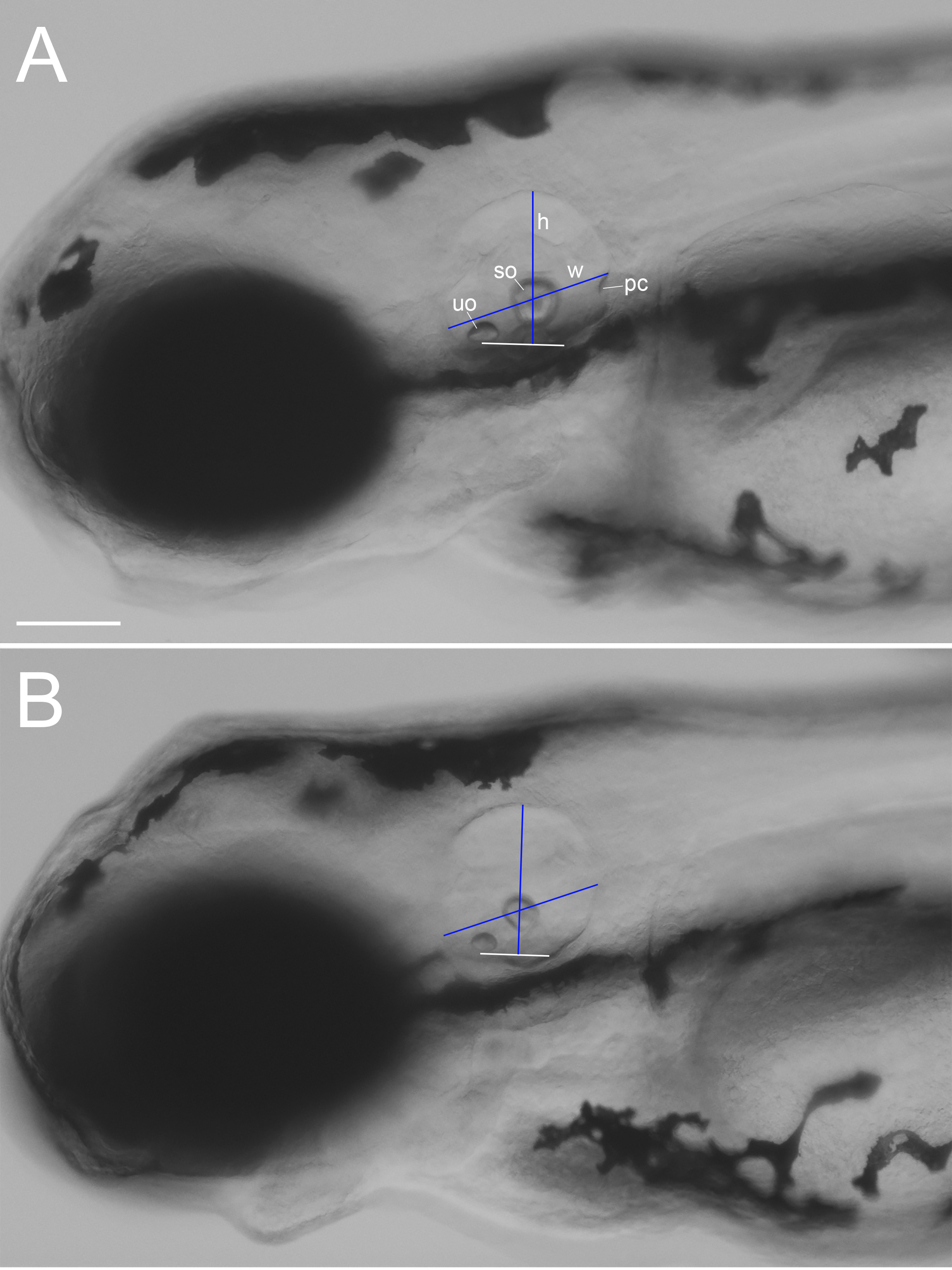

Supplement: Supplementary file 6 [file Image2.JPEG]

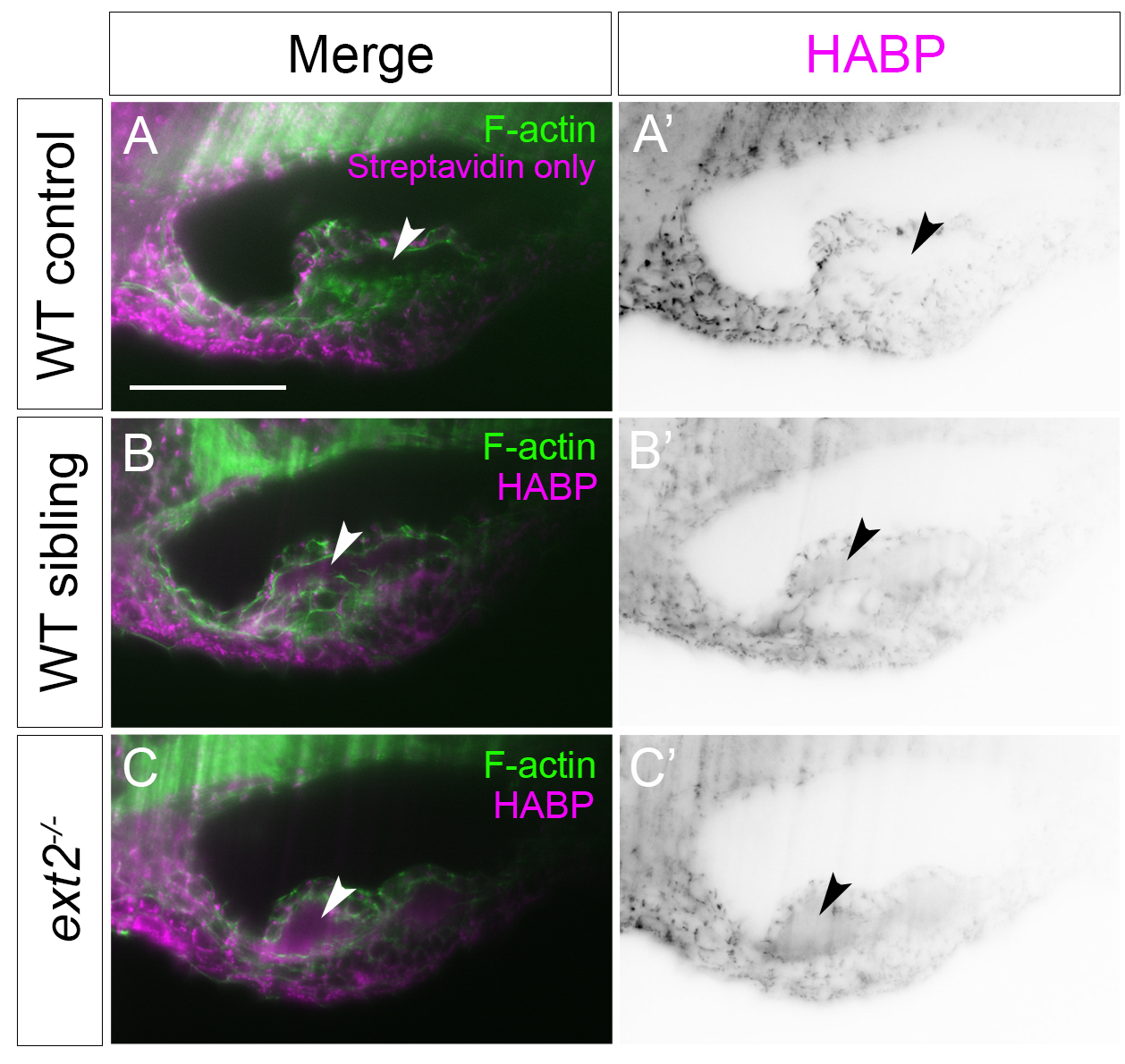

Supplement: Supplementary file 7 [file Image5.JPEG]
