# Supplementary material for: Presence of chondroitin sulphate and requirement for heparan sulphate biosynthesis in the developing zebrafish inner ear
Source: Front Cell Dev Biol. 2022 Aug 26;10:959624. doi: 10.3389/fcell.2022.959624 (PMC9458858; doi:10.3389/fcell.2022.959624)
Supplement: Supplementary file 8 [file DataSheet1.pdf]

Presence of chondroitin sulphate and requirement for heparan sulphate biosynthesis in the developing zebrafish inner ear

Ana A. Jones†, Elvira Diamantopoulou†, Sarah Baxendale and Tanya T. Whitfield*

Supplementary Figure and Movie Legends, and Supplementary Table

---

Supplementary Figure S1. Antibodies to chondroitin sulphate and keratan sulphate give distinct and reproducible staining patterns in the wild-type zebrafish ear

Antibody staining experiment from the same batch of embryos (phenotypically wild-type siblings from a cross between *ext2*$^{+/-}$ heterozygous parents), performed under the same conditions, with the same secondary antibody. **(A–A")** Phalloidin stain for F-actin (A) and antibody stain for chondroitin sulphate (A') at 48 hpf. Arrowheads mark small patches of stain associated with the outgrowing anterior epithelial projection (ep) and on the basal side of the epithelium where the ventral projection will emerge. **(B–B")** Phalloidin stain for F-actin (B) and antibody stain for keratan sulphate (B') at 48 hpf. Both antibodies mark the otolithic membrane (asterisk). Lateral views; anterior to the left, dorsal to the top. Abbreviations: CS, chondroitin sulphate; ep, anterior epithelial projection; KS, keratan sulphate; um, utricular macula; uo, utricular otolith. Scale bar in A, 50 µm (applies to all panels).

Supplementary Figure S2. Morphometric measurements of ear and otolith size in *ext2* mutants

Head of a phenotypically wild-type sibling embryo **(A)** and *ext2* homozygous mutant **(B)** at 72 hpf. Blue lines show measurements taken in Fiji of ear width (w) from the dorsal side of the posterior crista (pc), through the saccular otolith (so, sagitta) to the anterior of the ear. Ear height (h) was measured from a position level with the ventral side of the utricular otolith (uo; lapillus), shown by the horizontal white line, to the dorsal-most point of the ear. Otolith areas were traced from the same images in Fiji. Lateral views; anterior to the left. Scale bar in A, 100 µm.

Supplementary Figure S3. Expression of *ext2* in wild-type and *ext2* mutant embryos at 50 and 72 hpf

(A–H) In situ hybridisation to *ext2* in phenotypically wild-type sibling and *ext2* mutant embryos at 50 and 72 hpf. Expression in the wild type (A,C,E,G) was regionalised by 50 hpf, with highest levels in the head and brain, pectoral fin buds (black arrowheads), spinal cord (sc) and posterior lateral line neuromasts (nm), and lower expression in the heart and

somites. In *ext2* mutants (B,D,F,H), expression was present in a normal spatial pattern, but at reduced levels. Note reduced expression in the rudimentary fin buds at 50 hpf (B, white arrowhead). (G,H) Detailed view of the otic vesicle (outlined) at 72 hpf shows reduced expression of *ext2* in the *ext2* mutant ear. A,B, dorsal views; C–H, lateral views; all images are anterior to the left. Abbreviations: lp, lateral projection; nm, posterior lateral line neuromast; pc, posterior crista; um, utricular (anterior) macula. Scale bar in A, 200 µm (applies to A–D); in E, 200 µm (applies to E,F); in G, 50 µm (applies to G,H).

Supplementary Figure S4. Expression of *ugdh* is present, but reduced, in *ext2* mutant embryos, including in the ear

(A–D') In situ hybridisation to *ugdh* in phenotypically wild-type sibling and *ext2* mutant embryos. In wild-type embryos (A,C), expression is present throughout the brain, pectoral fin (pf) and in the ear (arrowhead; enlarged in A',C'). The box in A' is an overlay from a different focal plane to the main image, to show strong expression in the epithelial projections, including the ventral bulge from the lateral projection. In the *ext2* mutant (B,D), expression is present in a similar spatial pattern but levels appear reduced throughout, including in the ear (arrowhead; enlarged in B',D'). The eyes have been removed in C and D for ease of mounting. All images are lateral views with anterior to the left. Scale bar in A, 200 µm (applies to A–D); scale bar in A', 50 µm (applies to A'–D').

Supplementary Figure S5. HA is still present in the lateral projection of the *ext2* mutant ear

(A–C') Light-sheet fluorescence images of the lateral projection at 48 hpf. **(A, A')** Control sample (wild type, streptavidin only; no HABP). Non-specific background staining was present on the skin of control samples, but levels of fluorescence in the core of the lateral projection (arrowhead) were low. **(B–C')** In phenotypically wild-type sibling embryos (B,B') and in *ext2* mutants (C,C'), staining was present in the core of the lateral projection (arrowheads), highlighted in the single channel images (HABP, inverted grayscale). Dorsal views; anterior to the left. Scale bar in A, 50 µm (applies to all panels).

Supplementary Movie 1. Chondroitin sulphate staining in the wild-type ear at 48 hpf

A series of *z*-slices through a phenotypically wild-type sibling ear at 48 hpf stained with anti-chondroitin sulphate (magenta) and Alexa phalloidin for F-actin (green). Lateral view with anterior to the left; the movie plays from lateral to medial. The epidermis over the ear is strongly positive for CS. CS staining is present in the posterior bulge of the lateral projection and in a patch or streak beneath ventral otic epithelium. Weak staining in the cores of the anterior and lateral projections is visible towards the end of the movie. Note the lack of CS staining in the endolymphatic duct. Scale bar, 50 µm.

Supplementary Movie 2. Chondroitin sulphate staining in the *ext2* mutant ear at 48 hpf

A series of *z*-slices through an *ext2* mutant ear at 48 hpf stained with anti-chondroitin sulphate (magenta) and Alexa phalloidin for F-actin (green). The epidermis over the ear is strongly positive for CS. Strong CS staining is evident in the posterior bulge of the lateral projection, together with a discrete patch beneath ventral otic epithelium. Lateral view with anterior to the left; the movie plays from lateral to medial. Scale bar, 50 µm.

Supplementary Movie 3. Chondroitin sulphate staining in the wild-type ear at 65 hpf

A series of *z*-slices through a phenotypically wild-type sibling ear at 65 hpf stained with anti-chondroitin sulphate (magenta) and Alexa phalloidin for F-actin (green). The epidermis over the ear is strongly positive for CS. The projections in the ear have now fused to form three pillars, and there is very little CS staining visible in the cores of the pillars or lateral projection at this stage. CS staining is present in the otolithic membrane overlying hair cells of the utricular (anterior) macula. Lateral view with anterior to the left; the movie plays from lateral to medial. Scale bar, 50 µm.

Supplementary Movie 4. Chondroitin sulphate staining in the *ext2* mutant ear at 65 hpf

A series of *z*-slices through an *ext2* mutant ear at 65 hpf stained with anti-chondroitin sulphate (magenta) and Alexa phalloidin for F-actin (green). The epidermis over the ear is strongly positive for CS. The cores of the posterior bulge, posterior projection and unfused ventral projection are all strongly positive for CS. CS staining in the otolithic membrane is present, but weaker than in the wild type. Lateral view with anterior to the left; the movie plays from lateral to medial. Scale bar, 50 µm.

Supplementary Table 1. List of genes used to generate in situ hybridisation probes, with references of original characterisation

Gene	ZFIN ID	Reference
chsy1	ZDB-GENE-030131-3127	(Li et al., 2010)
ext2	ZDB-GENE-041124-3	(Söllner et al., 2003)
otog	ZDB-GENE-120228-1	(Stooke-Vaughan et al., 2015)
otomp	ZDB-GENE-040709-1	(Murayama et al., 2005)
stm	ZDB-GENE-031112-4	(Söllner et al., 2003)
tecta	ZDB-GENE-110411-120	(Stooke-Vaughan et al., 2015)
ugdh	ZDB-GENE-011022-1	(Busch-Nentwich et al., 2004)
vcanb (formerly *dermacan, cspg2b*)	ZDB-GENE-030131-2185	(Kang et al., 2004)
